# Assessing the relationships of 1,400 blood metabolites with abdominal aortic aneurysm: a Mendelian randomization study

**DOI:** 10.3389/fphar.2024.1514293

**Published:** 2025-01-03

**Authors:** Qian Guo, Xinghua Xu, Xiaohui Li, Yang Mao, Shengqiang Li, Yuxin Yao, Xiang Li, Yaxing Li, Jiayue Feng, Yan Shu, Xingli Xu

**Affiliations:** ^1^ Department of Rhinology, The First Affiliated Hospital of Zhengzhou University, Zhengzhou, China; ^2^ School of Basic Medicine, Shandong First Medical University & Shandong Academy of Medical Sciences, Jinan, China; ^3^ Department of Cardiology, State Key Laboratory for Innovation and Transformation of Luobing Theory, Key Laboratory of Cardiovascular Remodeling and Function Research, Chinese Ministry of Education, Chinese National Health Commission and Chinese Academy of Medical Sciences, Qilu Hospital of Shandong University, Jinan, China; ^4^ Department of Critical Care Medicine, Qilu Hospital of Shandong University, Jinan, China; ^5^ Department of Anesthesiology, The Second Hospital, Cheeloo College of Medicine, Shandong University, Jinan, China; ^6^ Department of Cardiology, Sichuan Provincial People’s Hospital, University of Electronic Science and Technology of China, Chengdu, China

**Keywords:** Mendelian randomization study, abdominal aortic aneurysm, metabolites, metabolite ratios, metabolomics

## Abstract

**Background:**

Abdominal aortic aneurysm (AAA) is one of the most dangerous types of vascular diseases worldwide. Metabolic disturbance affects disease risk and provide underlying therapeutic targets. Previous studies have reported an association between metabolic disorders and AAA. However, evidence of a causal relationship between blood metabolites and AAA is still lacking at present.

**Methods:**

Using Mendelian randomization (MR), we assessed the causal association between 1,400 serum metabolites and AAA. The inverse variance weighted method (IVW), weighted median, MR-Egger regression, simple mode, as well as weighted mode methods were used for evaluating the causality between blood metabolites and AAA. Pleiotropy and heterogeneity tests were further conducted.

**Results:**

Through strict screening, 17 known metabolites, 7 unknown metabolites and 5 metabolite ratios related to AAA were identified. Among all the metabolites, 24 were found to have negative associations, while 5 exhibited positive associations. The top five metabolites associated with an increased risk of AAA were Oleoyl-linoleoyl-glycerol (18:1/18:2) [2], Glycosyl-N-(2-hydroxynervonoyl)-sphingosine (d18:1/24:1(2OH)), Glycochenodeoxycholate 3-sulfate, X-21441 and X-24328. In contrast, the top five metabolites that were linked to a reduced risk of AAA included Uridine to pseudouridine ratio, Octadecanedioate, Phosphate to oleoyl-linoleoyl-glycerol (18:1 to 18:2) [2] ratio, 1-(1-enyl-palmitoyl)-GPE (p-16:0), and 1-stearoyl-GPG (18:0).

**Conclusion:**

Among the 1,400 blood metabolites, we identified 17 known metabolites, 7 unknown metabolites, and 5 metabolite ratios associated with AAA. This MR study may provide a novel significant insight for the screening and prevention of AAA.

## 1 Introduction

Abdominal aortic aneurysm (AAA) indicates as the maximal localized dilation of the abdominal aorta with the diameter ≥30 mm or 1.5 times greater than normal (Lu et al., 2022). The computed tomography (CT), magnetic resonance imaging (MRI) and ultrasound for aortic imaging are acceptable and reliable methods for early detection of AAA (Sakalihasan et al., 2018). AAA leads to about 200,000 deaths, including 9% in men over 65 years of age each year worldwide. The proven risk factors for AAA include male sex, age, smoking, hypercholesterolemia, hyperlipidemia, and hypertension with high heritability (Sakalihasan et al., 2018). Currently, surgical interventions including open aneurysm repair or endovascular aneurysm repair (EVAR) are limited options for patients with AAA larger than 5.5 cm in diameter (Vanmaele et al., 2024). Its high mortality is mainly due to the clinical lack of reliable and effective drugs treatment. The value of stains, β-blockers, antibiotics, or anti-platelet therapy in reducing the progression of AAA still needs further investigation (Zhang et al., 2024).

Metabolites are intermediates or end products of metabolic reactions. Their levels are affected by genetics, lifestyle, diet, gut microbiota and diseases. Besides, they can further influence disease conditions and being potential therapeutic targets. Recently, several studies have found that the plasma metabolites may be underlying biomarkers to explore the diagnosis and prognosis of AAA, as well as targets for alleviating the pathological progression of AAA (Tian et al., 2022; Benson et al., 2023; Li et al., 2024). Metabolic changes in patients with AAA are primarily related to carbohydrate and lipid metabolism, insulin resistance, energy metabolism, and alterations in amino acid (AA) metabolism (Li et al., 2024; Ling et al., 2022; Lieberg et al., 2021).

Due to hereditary variations in certain metabolite levels, human genetics can be utilized to assess the role of metabolites in disease outcomes. Mendelian randomization (MR) is a causal inference method that uses single nucleotide polymorphisms (SNPs) occurring randomly in the human genome as instrumental variables to test the impact of exposure, such as metabolites on disease outcomes (Wang et al., 2023; Chen et al., 2023). Currently, there is a lack of cohort-based causal studies linking metabolites to AAA. Therefore, this study elucidates changes in metabolite expression levels in AAA and their effects through comprehensive blood metabolomics data collection and MR analysis, providing new reliable targets for AAA diagnosis and treatment.

## 2 Methods

### 2.1 Experimental design

The complete dataset in this study is publicly accessible from published genome-wide association studies (GWAS) as reported on the database website. Written informed consent was individually obtained from all participants under approval from Institutional Review Boards’ ethics committees. No further ethical approval or informed consent is necessary. We conducted a comprehensive assessment using MR to investigate the relationships between metabolites as exposures and AAA as the outcome. A total of 1,400 metabolite, including 1,091 metabolites and 309 metabolite ratios was incorporated. The clinical diagnosis of AAA was determined as a localized dilatation of the abdominal aorta to a diameter ≥3.0 cm (outer wall to outer wall), as measured by imaging techniques such as using CT, MRI or ultrasound (Lu et al., 2022; Sakalihasan et al., 2018).

**FIGURE 1:**
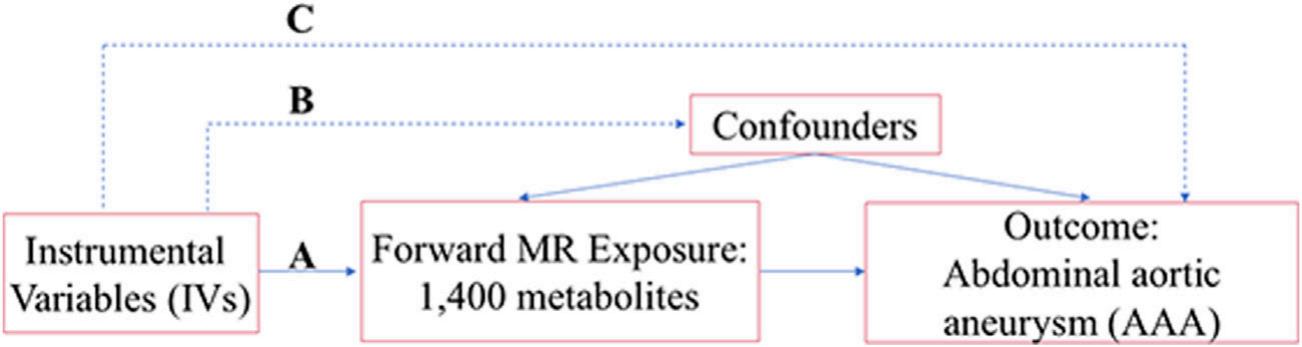
The schematic diagram of the MR study.

Three critical assumptions are necessary for fully consideration during the scientific MR studies. A), Genetic instruments should strongly correlate with the exposure under study. B), Genetic instrumental variables are not influenced by any known or unknown confounding factors and independent of the outcome. C), The instrumental variables should influence the outcome only through their impacts on the exposure of interests. Therefore, this dataset was used to investigate the role of 1,400 metabolites in patients diagnosed with AAA and healthy individuals without AAA using a MR analysis approach. The schematic illustration of this MR study is shown in Figure 1.

### 2.2 Data sources

The Canadian Longitudinal Study on Aging (CLSA) is a large-scale and long-term research, designed to investigate and track the health status and life transitions in Canada over many years. It aims to collect various aspects of participants’ lives for evaluating the factors that contribute to healthy aging and the development of age-related diseases. Chen et al. performed the GWAS, involving metabolomic data of 1,091 metabolites and 309 metabolite ratios, from a cohort of 8,299 participants belonging to CLSA dataset. The results of this GWAS could be accessible including detailed human plasma metabolomic data from the website http://metabolomics.helmholtzmuenchen.de/gwas/.

The United Kingdom (UK) Biobank is a large-scale biomedical research resource, including around 500,000 participants aged 40–69 years between 2006 and 2010, established in the UK. It concludes detailed health information, lifestyle, donated biological samples, genome-wide genotyping, results of imaging and medical records over a period of decades. We defined AAA in the UK Biobank dataset based on the electronic health recodes (ICD-9/10 diagnosis and hospital procedure codes) from hospital episode statistics and death certificates. Age, sex, principal components and genotyping batch were all adjusted in the analysis. A total of 3,658 patients with AAA and 244,907 controls without AAA was included. The results of this statistics for AAA were obtained from Pan-UK Biobank service https://pan.ukbb.broadinstitute.org/.

### 2.3 Independent variable selection

In the MR analysis, the core assumptions refer to the fundamental principles that underpin the effectiveness of using genetic variants as proxies for modifiable exposures. Genetic variants as instrumental variables (IV) are associated with the environmental exposure as risk factors, independent of confounding factors common in traditional observational studies. Three core assumptions should be followed in the IV selection to avoid the biased estimates in MRandomization studies, including relevance (instrument-relevance assumption), independence (instrument-independence assumption) and exclusion restriction (no pleiotropy). We established a threshold of *p* < 5*10^–8^ of each metabolite for identifying SNPs that exhibit significant association on a genome-wide scale. Pairs of SNPs were considered to exhibit significant linkage disequilibrium if the squared correlation coefficient (r^2) was less than 0.1, and if the SNPs were located within a 500-kilobase (kb) genomic radius (Yang et al., 2020). Additionally, SNPs with an F-statistic below 10 were categorized as weak instruments and underwent rigorous scrutiny to minimize bias arising from weak instrumentality (Choi et al., 2019).

### 2.4 Sensitivity analysis

MR sensitivity analysis is a method performed to assess the robustness of causal inference, testing the potential violating assumptions or unmeasured confounding factors such as age, sex, and lifestyle variables. Researchers typically assess these assumptions rigorously through sensitivity analyses and by considering alternative explanations for their findings. Specifically, employing the Inverse Variance Weighted (IVW) method was used for assessing the causal relationship between metabolites and AAA, as the cornerstone of this analysis (Zhao and Liu, 2024). MR-Egger and the Weighted Median (WM) were further performed as the secondary methods of evaluation. First, Cochran’s Q test was conducted using both the IVW and MR-Egger methods to detect potential violations of assumptions due to heterogeneity in IV correlations. Second, the MR-Egger intercept was then utilized to assess pleiotropy, ensuring that genetic variants are independently associated with both metabolites and AAA. Third, we employed WM and Mode-based Estimation to enhance the reliability and stability of our hypothesis testing. Last, individual SNP analyses and leave-one-out (LOO) diagnostics were conducted to assess the robustness of observed associations for each SNP. The MR analysis assumes causality under the condition of genetic correlation between metabolites as exposure and AAA as outcome. To mitigate bias, SNPs associated with aneurysms were carefully selected, excluding those linked to other traits. Nonetheless, SNPs lacking known associations may still influence the incidence of AAA.

### 2.5 Statistical methods

MR analyses were conducted using the ‘Two Sample MR’ package in R software (version 4.2.1). Odds ratios (ORs) were utilized to assess the magnitude and direction of the metabolic impact, accompanied by their respective 95% confidence intervals (CIs).

## 3 Results

Through strict screening, 17 known metabolites, 7 unknown metabolites and 5 metabolite ratios related to AAA were identified. Among all metabolites, 24 were found to have negative associations, and 5 had positive associations. The top five metabolites that increased the risk of AAA were Oleoyl-linoleoyl-glycerol (18:1/18:2) [2], Glycosyl-N-(2-hydroxynervonoyl)-sphingosine (d18:1/24:1(2OH)), Glycochenodeoxycholate 3-sulfate, X-21441 and X-24328. Conversely, the top five metabolites that decreased the risk of AAA were Uridine to pseudouridine ratio, Octadecanedioate, Phosphate to oleoyl-linoleoyl-glycerol (18:1 to 18:2) [2] ratio, 1-(1-enyl-palmitoyl)-GPE (p-16:0), and 1-stearoyl-GPG (18:0).

Based on the threshold of *p* < 5*10^–8^ of each metabolite for identifying SNPs with significant associations on a genome-wide scale, a total of 29 plasma metabolites and metabolite ratios were selected. All computed F-statistics exceeded 10, indicating minimal susceptibility to weak instrument bias. All metabolic analyses utilized the IVW method as the primary approach, demonstrating uniformity and robust instrument strength. After screening for the primary outcomes and pleiotropy, 29 metabolites related to AAA were identified with IVW *p* < 0.05 and Pleiotropy *p* > 0.05, including 24 metabolites and 5 metabolite ratios.

**FIGURE 2:**
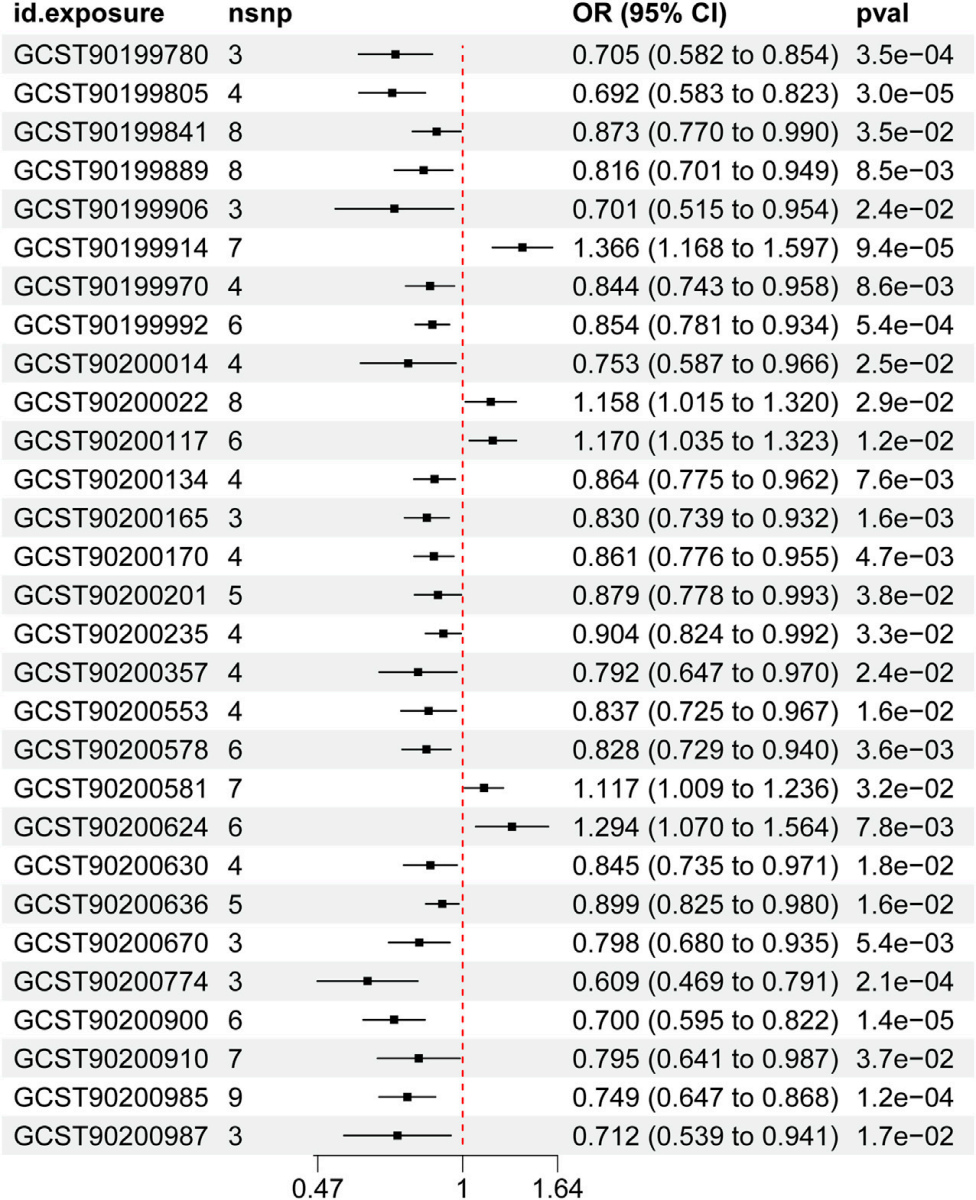
IVW forest maps of 29 significantly related metabolites.

**FIGURE 3:**
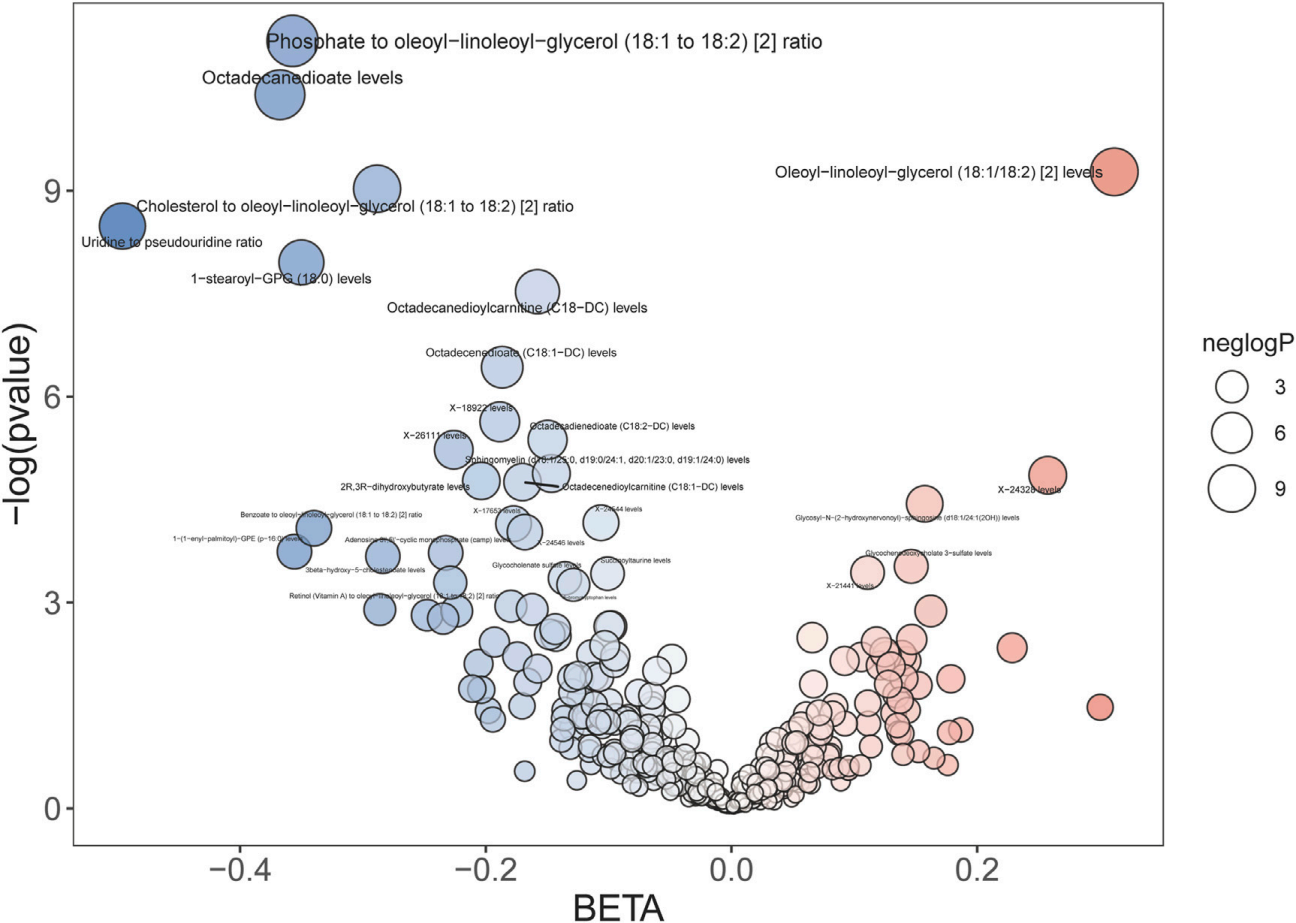
Bubble plot of 29 significantly related metabolites.

Among the 24 metabolites, the chemical properties of 7 metabolites are unknown, while the remaining 17 known metabolites belong to multiple categories, such as lipid metabolism, bile acid metabolism, ketone body metabolism, and sphingolipid metabolism. Remarkably, lipids metabolism constitutes the most prevalent category, comprising approximately 60% of the identified substances. Within the group of 22 metabolites and metabolite ratios, only 3 known metabolites show a positive association with AAA, while the other 14 metabolites and 5 metabolite ratios are negative associated with the condition. The IVW forest plot depicting the association of 29 significantly associated metabolites and metabolite ratios is shown in Figure 2. The bubble plot is further performed to represent visually the relationships between the metabolites and metabolite rations and AAA as shown in Figure 3. Detailed results of alternative MR analyses, Q-tests, and sensitivity analyses for the 29 identified metabolites are provided in Table 1. All instrumental variables (IVs) passed rigorous sensitivity tests (*p* > 0.05).

**TABLE 1:** Two MR models assessed causal relationships between 29 metabolites, their ratios, and AAA, examining heterogeneity and potential pleiotropy.

Class	Metabolite name and number	Method	SNP(n)	pval	OR	95%CI	Heterogeneity		Pleiotropy	
							Qvalue	P	Intercept	P
Lipid	GCST90199780	Inverse variance weighted	3	3.5e-04	0.705	0.582–0.854	1.431	0.489		
	1-stearoyl-GPG (18:0) levels	MR Egger	3	3.5e-04	0.705	0.582–0.854	0.697	0.404	0.044	0.549
Lipid	GCST90199805	Inverse variance weighted	4	3.0e-05	0.692	0.583–0.823	0.515	0.916		
	Octadecanedioate levels	MR Egger	4	3.0e-05	0.692	0.583–0.823	0.327	0.849	0.022	0.707
Bile acid	GCST90199841	Inverse variance weighted	8	3.5e-02	0.873	0.770–0.990	18.989	0.008		
	Glycocholenate sulfate levels	MR Egger	8	3.5e-02	0.873	0.770–0.990	18.966	0.004	−0.003	0.935
Ketone body	GCST90199889	Inverse variance weighted	8	8.5e-03	0.816	0.701–0.949	10.574	0.158		
	2R,3R-dihydroxybutyrate levels	MR Egger	8	8.5e-03	0.816	0.701–0.949	8.486	0.205	−0.032	0.27
Lipid	GCST90199906	Inverse variance weighted	3	2.4e-02	0.701	0.515–0.954	1.112	0.574		
	1-(1-enyl-palmitoyl)-GPE (p-16:0) levels	MR Egger	3	2.4e-02	0.701	0.515–0.954	0.047	0.829	0.265	0.49
Lipid	GCST90199914	Inverse variance weighted	7	9.4e-05	1.366	1.168–1.597	7.098	0.312		
	Oleoyl-linoleoyl-glycerol (18:1/18:2) [2] levels	MR Egger	7	9.4e-05	1.366	1.168–1.597	6.614	0.251	−0.018	0.572
Lipid	GCST90199970	Inverse variance weighted	4	8.6e-03	0.844	0.743–0.958	5.579	0.134		
	Octadecenedioylcarnitine (C18:1-DC) levels	MR Egger	4	8.6e-03	0.844	0.743–0.958	4.597	0.1	−0.023	0.581
Lipid	GCST90199992	Inverse variance weighted	6	5.4e-04	0.854	0.781–0.934	4.683	0.456		
	Octadecanedioylcarnitine (C18-DC) levels	MR Egger	6	5.4e-04	0.854	0.781–0.934	4.675	0.322	0.002	0.939
Lipid	GCST90200014	Inverse variance weighted	4	2.5e-02	0.753	0.587–0.966	11.276	0.01		
	3beta-hydroxy-5-cholestenoate levels	MR Egger	4	2.5e-02	0.753	0.587–0.966	4.501	0.105	−0.098	0.225
Bile acid	GCST90200022	Inverse variance weighted	8	2.9e-02	1.158	1.015–1.320	7.047	0.424		
	Glycochenodeoxycholate 3-sulfate levels	MR Egger	8	2.9e-02	1.158	1.015–1.320	3.672	0.721	0.039	0.116
Sphingolipid	GCST90200117	Inverse variance weighted	6	1.2e-02	1.170	1.035–1.323	3.747	0.586		
	Glycosyl-N-(2-hydroxynervonoyl)-sphingosine (d18:1/24:1(2OH)) levels	MR Egger	6	1.2e-02	1.170	1.035–1.323	1.342	0.854	−0.036	0.196
Lipid	GCST90200134	Inverse variance weighted	4	7.6e-03	0.864	0.775–0.962	1.803	0.614		
	Sphingomyelin (d18:1/25:0, d19:0/24:1, d20:1/23:0, d19:1/24:0) levels	MR Egger	4	7.6e-03	0.864	0.775–0.962	0.626	0.731	0.026	0.391
Lipid	GCST90200165	Inverse variance weighted	3	1.6e-03	0.830	0.739–0.932	2.482	0.289		
	Octadecenedioate (C18:1-DC) levels	MR Egger	3	1.6e-03	0.830	0.739–0.932	2.354	0.125	−0.013	0.854
Lipid	GCST90200170	Inverse variance weighted	4	4.7e-03	0.861	0.776–0.955	4.1	0.251		
	Octadecadienedioate (C18:2-DC) levels	MR Egger	4	4.7e-03	0.861	0.776–0.955	3.82	0.148	−0.018	0.739
Metabolites	GCST90200201	Inverse variance weighted	5	3.8e-02	0.879	0.778–0.993	3.336	0.503		
	6-bromotryptophan levels	MR Egger	5	3.8e-02	0.879	0.778–0.993	0.992	0.803	−0.061	0.223
Metabolites	GCST90200235	Inverse variance weighted	4	3.3e-02	0.904	0.824–0.992	1.956	0.581		
	Succinoyltaurine levels	MR Egger	4	3.3e-02	0.904	0.824–0.992	1.947	0.378	0.003	0.931
Metabolites	GCST90200357	Inverse variance weighted	4	2.4e-02	0.792	0.647–0.970	3.098	0.377		
	Adenosine 3\',5\'-cyclic monophosphate (camp) levels	MR Egger	4	2.4e-02	0.792	0.647–0.970	0.352	0.838	0.09	0.239
Unknown	GCST90200553	Inverse variance weighted	4	1.6e-02	0.837	0.725–0.967	3.117	0.374		
	X-17653 levels	MR Egger	4	1.6e-02	0.837	0.725–0.967	1.401	0.496	0.055	0.32
Unknown	GCST90200578	Inverse variance weighted	6	3.6e-03	0.828	0.729–0.940	2.433	0.787		
	X-18922 levels	MR Egger	6	3.6e-03	0.828	0.729–0.940	1.185	0.881	0.039	0.327
Unknown	GCST90200581	Inverse variance weighted	7	3.2e-02	1.117	1.009–1.236	1.792	0.938		
	X-21441 levels	MR Egger	7	3.2e-02	1.117	1.009–1.236	1.7	0.889	0.009	0.774
Unknown	GCST90200624	Inverse variance weighted	6	7.8e-03	1.294	1.070–1.564	0.258	0.998		
	X-24328 levels	MR Egger	6	7.8e-03	1.294	1.070–1.564	0.205	0.995	0.009	0.83
Unknown	GCST90200630	Inverse variance weighted	4	1.8e-02	0.845	0.735–0.971	3.495	0.321		
	X-24546 levels	MR Egger	4	1.8e-02	0.845	0.735–0.971	1.066	0.587	0.06	0.259
Unknown	GCST90200636	Inverse variance weighted	5	1.6e-02	0.899	0.825–0.980	2.59	0.629		
	X-24544 levels	MR Egger	5	1.6e-02	0.899	0.825–0.980	0.456	0.928	−0.028	0.24
Unknown	GCST90200670	Inverse variance weighted	3	5.4e-03	0.798	0.680–0.935	1.548	0.461		
	X-26111 levels	MR Egger	3	5.4e-03	0.798	0.680–0.935	0.061	0.805	0.044	0.437
Ratio	GCST90200774	Inverse variance weighted	3	2.1e-04	0.609	0.469–0.791	0.071	0.965		
	Uridine to pseudouridine ratio	MR Egger	3	2.1e-04	0.609	0.469–0.791	0.024	0.876	−0.042	0.865
Ratio	GCST90200900	Inverse variance weighted	6	1.4e-05	0.700	0.595–0.822	5.307	0.38		
	Phosphate to oleoyl-linoleoyl-glycerol (18:1 to 18:2) [2] ratio	MR Egger	6	1.4e-05	0.700	0.595–0.822	5.282	0.26	0.004	0.897
Ratio	GCST90200910	Inverse variance weighted	7	3.7e-02	0.795	0.641–0.987	12.694	0.048		
	Retinol (Vitamin A) to oleoyl-linoleoyl-glycerol (18:1 to 18:2) [2] ratio	MR Egger	7	3.7e-02	0.795	0.641–0.987	6.463	0.264	0.06	0.08
Ratio	GCST90200985	Inverse variance weighted	9	1.2e-04	0.749	0.647–0.868	6.207	0.624		
	Cholesterol to oleoyl-linoleoyl-glycerol (18:1 to 18:2) [2] ratio	MR Egger	9	1.2e-04	0.749	0.647–0.868	3.681	0.816	0.041	0.156
Ratio	GCST90200987	Inverse variance weighted	3	1.7e-02	0.712	0.539–0.941	3.984	0.136		
	Benzoate to oleoyl-linoleoyl-glycerol (18:1 to 18:2) [2] ratio	MR Egger	3	1.7e-02	0.712	0.539–0.941	2.383	0.123	0.058	0.563

Among the 17 identified metabolites, we found that Octadecanedioate levels have the most significant negative correlation with AAA (IVW OR = 0.692, 95% CI = 0.583–0.823, *p* < 0.001), followed by 1-(1-enyl-palmitoyl)-GPE (p-16:0) levels (IVW OR = 0.701, 95% CI = 0.515–0.954, *p* = 0.024), 1-stearoyl-GPG (18:0) levels (IVW OR = 0.705, 95% CI = 0.582–0.854, *p* < 0.001), 2-linoleoylglycerol (18:2) levels (IVW OR = 0.96, 95% CI = 0.94–0.99, *p* = 0.003), 3beta-hydroxy-5-cholestenoate levels (IVW OR = 0.753, 95% CI = 0.587–0.966, *p* = 0.025), 2R,3R-dihydroxybutyrate levels (IVW OR = 0.816, 95% CI = 0.701–0.949, *p* < 0.001), Octadecenedioate (C18:1-DC) levels (IVW OR = 0.830, 95% CI = 0.739–0.932, *p* < 0.001), Octadecenedioylcarnitine (C18:1-DC) levels (IVW OR = 0.844, 95% CI = 0.743–0.958, *p* < 0.001), Octadecanedioylcarnitine (C18-DC) levels (IVW OR = 0.854, 95% CI = 0.781–0.934, *p* < 0.001), Octadecadienedioate (C18:2-DC) levels (IVW OR = 0.861, 95% CI = 0.776–0.955, *p* < 0.001), Sphingomyelin (d18:1/25:0, d19:0/24:1, d20:1/23:0, d19:1/24:0) levels (IVW OR = 0.864, 95% CI = 0.775–0.962, *p* < 0.001), Glycocholenate sulfate levels (IVW OR = 0.873, 95% CI = 0.770–0.990, *p* = 0.035), 6-bromotryptophan levels (IVW OR = 0.879, 95% CI = 0.778–0.993, *p* = 0.038), and Succinoyltaurine levels (IVW OR = 0.904, 95% CI = 0.824–0.992, *p* = 0.033).

The most significantly known positive correlation with AAA was observed in the Oleoyl-linoleoyl-glycerol (18:1/18:2) [2] levels (IVW OR = 1.366, 95% CI = 1.168–1.597, *p* < 0.001), Glycosyl-N-(2-hydroxynervonoyl)-sphingosine (d18:1/24:1(2OH)) levels (IVW OR = 1.170, 95% CI = 1.035–1.323; *p* = 0.012), and Glycochenodeoxycholate 3-sulfate levels (IVW OR = 1.158, 95% CI = 1.015–1.32, *p* = 0.029).

In relation to metabolite ratios, a collective of 5 ratios all exhibit a negative correlation with AAA. Among them, the most significant negative correlation with AAA was observed in the ratio of Uridine to pseudouridine (IVW OR = 0.609, 95% CI = 0.469–0.791, *p* < 0.001), followed by Phosphate to oleoyl-linoleoyl-glycerol (18:1 to 18:2) [2] ratio (IVW OR = 0.7, 95% CI = 0.595–0.822, *p* < 0.001), Benzoate to oleoyl-linoleoyl-glycerol (18:1 to 18:2) [2] ratio (IVW OR = 0.712, 95% CI = 0.539–0.941, *p* = 0.017), Cholesterol to oleoyl-linoleoyl-glycerol (18:1 to 18:2) [2] ratio (IVW OR = 0.749, 95% CI = 0.647–0.868, *p* < 0.001), Retinol (Vitamin A) to oleoyl-linoleoyl-glycerol (18:1 to 18:2) [2] ratio (IVW OR = 0.795, 95% CI = 0.641–0.987, *p* = 0.037).

In summary, MR estimates from IVW, WM, and MR-Egger regression models across 24 metabolites and 5 metabolite ratios consistently indicated both direction and magnitude, thereby bolstering the robustness of causal inference, with the exception of 3beta-hydroxy-5-cholestenoate levels (IVW OR = 0.753, 95% CI = 0.587–0.966, *p* = 0.025, heterogeneity Q value = 11.276; *p* = 0.01) and Retinol (Vitamin A) to oleoyl-linoleoyl-glycerol (18:1 to 18:2) [2] ratio (IVW OR = 0.795, 95% CI = 0.641–0.987, *p* = 0.037, heterogeneity Q value = 12.94, *p* = 0.048). No significant heterogeneity was observed in the p-values from the Cochran Q test across the remaining metabolites and metabolite ratios (Table 1). The MR-Egger intercept did not suggest the presence of pleiotropy (Table 1). Additionally, a LOO analysis did not reveal any highly influential SNPs that could bias the aggregated effect estimates (Supplementary Figures S1–S29). The funnel plot for the distribution of SNPs, scatter plot for the causal effect and forest plot of single SNP MR were also shown in (Supplementary Figures S1–S29. Consequently, these 24 metabolites and 5 metabolite ratios are identified as potential candidate markers in the metabolomic profile associated with the pathogenesis of AAA.

## 4 Discussion

Our research findings substantiate a causal association between 24 metabolites and 5 metabolite ratios with AAA. The results indicate potential causal links between circulating metabolites and AAA. Specifically, increased levels of Oleoyl-linoleoyl-glycerol (18:1/18:2) [2], Glycosyl-N-(2-hydroxynervonoyl)-sphingosine (d18:1/24:1(2OH)), and Glycochenodeoxycholate 3-sulfate, exhibit a protective role in patients with AAA. Conversely, the elevation of 19 other metabolites and metabolite ratios including Octadecanedioate levels, 1-(1-enyl-palmitoyl)-GPE (p-16:0) levels, 1-stearoyl-GPG (18:0) levels, 2-linoleoylglycerol (18:2) levels, 3beta-hydroxy-5-cholestenoate levels, 2R,3R-dihydroxybutyrate levels, Octadecenedioate (C18:1-DC) levels, Octadecenedioylcarnitine (C18:1-DC) levels, Octadecanedioylcarnitine (C18-DC) levels, Octadecadienedioate (C18:2-DC) levels, Sphingomyelin (d18:1/25:0, d19:0/24:1, d20:1/23:0, d19:1/24:0) levels, Glycocholenate sulfate levels, 6-bromotryptophan levels, and Succinoyltaurine levels, Uridine to pseudouridine ratio, followed by Phosphate to oleoyl-linoleoyl-glycerol (18:1 to 18:2) [2] ratio (IVW OR = 0.7, 95% CI = 0.595–0.822, *p* = 0.000014), Benzoate to oleoyl-linoleoyl-glycerol (18:1 to 18:2) [2] ratio, Cholesterol to oleoyl-linoleoyl-glycerol (18:1 to 18:2) [2] ratio, as well as Retinol (Vitamin A) to oleoyl-linoleoyl-glycerol (18:1 to 18:2) [2] ratio, are associated with adverse effects on AAA.

AAA is a critical health concern that influences individuals throughout their entire lives (Lv et al., 2024a). It is characterized by pathological changes such as the loss of smooth muscle cells, alterations in extracellular matrix components, and significant inflammatory cell infiltration, which compromise arterial wall integrity (Xu et al., 2019). Additionally, metabolic homeostasis is disrupted, leading to altered serum concentrations of lipids, with elevated total cholesterol (TC), triglycerides and low-density lipoprotein cholesterol (LDL-C) as well as reduced high-density lipoprotein cholesterol (HDL-C) and phosphatidylcholines (Harrison et al., 2018). These changes lead to vascular dilation, increased wall stress, and an elevated risk of rupture, underscoring the importance of its early detection and monitoring (Golledge et al., 2023). Clinically, aneurysms frequently remain asymptomatic, but patients may report abdominal or back pain, a noticeable pulsatile mass, and fluctuations in blood pressure when symptoms do occur. Life-threatening complications, such as rupture, can result in severe internal bleeding, highlighting the importance of routine monitoring and imaging for those at higher risk (Golledge et al., 2023; Chen Z. et al., 2024). Patients with AAA are often complicated with atherosclerosis, hypertension, diabetes, and coronary artery disease, which can exacerbate its progression. Early identification and management of these comorbidities are further essential for reducing AAA risk and improving overall health outcomes.

The pathological process and progression of AAA are associated with lipid levels, particularly elevated LDL-C, which plays a significant role in atherosclerosis, a major contributor to AAA formation (Nana et al., 2021). Effective management of dyslipidemia may help mitigate AAA progression and enhance cardiovascular health, underscoring the necessity for routine lipid monitoring in at-risk individuals (Burillo et al., 2015). To our knowledge, this is the first investigation employing a MR approach to investigate the causal relationship between 1,400 blood metabolites and the risk of AAA.

After removing the unknown metabolites and metabolite ratios, we identified 19 metabolites and metabolite ratios that decreased the risk of AAA, including: Octadecanedioate, 1-(1-enyl-palmitoyl)-GPE (p-16:0), 1-stearoyl-GPG (18:0), 2-linoleoylglycerol (18:2), 3beta-hydroxy-5-cholestenoate, 2R,3R-dihydroxybutyrate, Octadecenedioate (C18:1-DC), Octadecenedioylcarnitine (C18:1-DC), Octadecanedioylcarnitine (C18-DC), Octadecadienedioate (C18:2-DC), Sphingomyelin (d18:1/25:0, d19:0/24:1, d20:1/23:0, d19:1/24:0), Glycocholenate sulfate, 6-bromotryptophan, and Succinoyltaurine, Uridine to pseudouridine ratio, followed by Phosphate to oleoyl-linoleoyl-glycerol (18:1 to 18:2) [2] ratio, Benzoate to oleoyl-linoleoyl-glycerol (18:1 to 18:2) [2] ratio, Cholesterol to oleoyl-linoleoyl-glycerol (18:1 to 18:2) [2] ratio, as well as Retinol (Vitamin A) to oleoyl-linoleoyl-glycerol (18:1 to 18:2) [2] ratio. Currently, no relevant studies have been found regarding 1-stearoyl-GPG (18:0), 3beta-hydroxy-5-cholestenoate, 2R,3R-dihydroxybutyrate, Octadecenedioylcarnitine (C18:1-DC), Sphingomyelin (d18:1/25:0, d19:0/24:1, d20:1/23:0, d19:1/24:0), Phosphate to oleoyl-linoleoyl-glycerol (18:1 to 18:2) [2] ratio, Benzoate to oleoyl-linoleoyl-glycerol (18:1 to 18:2) [2] ratio, and Retinol (Vitamin A) to oleoyl-linoleoyl-glycerol (18:1 to 18:2) [2] ratio. Lower levels of Octadecanedioate are related to the decreased odds of both preeclampsia and coronary heart disease (CHD) (Ross et al., 2019; Feofanova et al., 2020). Genes related to Octadecanedioate are significantly involved in the process of apoptosis (programmed cell death) based on the DAVID analysis (Feofanova et al., 2020). Different from the above two situations, exposure to Octadecanedioate downregulates the risk of AAA due to our MR results. High level of 1-(1-enyl-palmitoyl)-GPE (p-16:0) may be correlated with increased gastric cancer risk as a potential risk biomarker (Shu et al., 2021). 2-linoleoylglycerol (18:2) is one of reported circulating metabolome associated with colorectal cancer (CRC) (Gao et al., 2022). High level of Octadecenedioate (C18:1-DC) decreased susceptibility to CHD (Chen H. et al., 2024). Besides, its generation could be potentially decreased in patients with rosacea (Yao et al., 2024). Octadecanedioylcarnitine (C18-DC) mediates the genetic predictive effects on Alzheimer’s disease risk (Chen G. et al., 2024). Octadecadienedioate (C18:2-DC) is one of the plasma metabolites as significant mediators in the relationships between gut microbiota and type 2 diabetes (Zheng et al., 2024). Glycocholenate sulfate may be a new metabolite significantly associated with atrial fibrillation (AF), CHD, and CRC (Chen H. et al., 2024; Alonso et al., 2019; Alonso et al., 2015). Its concentration is negative associated with consumption of artificially sweetened beverages (Jia et al., 2024). 6-bromotryptophan may serve as a correlated metabolomics maker of kidney health, CKD progression, patients with cirrhosis, responder (Sekula et al., 2020; Tin et al., 2018; Sanchez et al., 2024). A MR study found a negative causal relationship with Succinoyltaurine and the risk of breast cancer (Ming et al., 2024). Uridine to pseudouridine ratio is related to decreased the risk of ischemia stroke (He et al., 2024). Sodium-glucose cotransporter 1 (SGLT1) and SGLT2 inhibitors play protective roles in small vessel disease (SVD) via Cholesterol to oleoyl-linoleoyl-glycerol (18:1 to 18:2) [2] ratio (Lv et al., 2024b). The upregulation of these metabolites and metabolite ratios may serve as good indicators for the occurrence and progression of AAA, while their upregulation could exert a protective effect. The metabolites are underlying diagnosis biomarkers and treatment targets for AAA. Given the correlation between these metabolites and AAA, it may be recommended to include clinical practice guidance to explore the changes in these above metabolites. Their downregulation may indicate early detection and development of AAA, and clinicians should pay attention to these metabolic markers for diagnosis and intervention.

After removing the unknown metabolites and metabolite ratios, we identified only 3 known metabolites that increased the risk of AAA, including: Oleoyl-linoleoyl-glycerol (18:1/18:2) [2], Glycosyl-N-(2-hydroxynervonoyl)-sphingosine (d18:1/24:1(2OH)), and Glycochenodeoxycholate 3-sulfate. Currently, no relevant studies have been found regarding Glycosyl-N-(2-hydroxynervonoyl)-sphingosine (d18:1/24:1(2OH)). Oleoyl-linoleoyl-glycerol (18:1/18:2) is a type of diacylglycerol that enhances the production and release of HDL-C and reduces the levels of TC and LDL-C by promoting the clearance of LDL-C and inhibiting cholesterol synthesis (Lv et al., 2024b). Glycochenodeoxycholate 3-sulfate, also named GCDCA-S, is produced from Glycochenodeoxycholic acid (GCDCA) in hepatocytes by sulfotransferase, reabsorbed in the distal small intestine, and taken up again by hepatocytes via Oatps, completing the enterohepatic circulation of bile acids (Li and Dawson, 2019). It helps regulate cholesterol metabolism by potentially reducing serum cholesterol levels and facilitates the fat digestion and fat-soluble vitamins absorption. GCDCA-S has been proved to be underlying diagnosis biomarkers for tuberculosis, severity of patients with acute hepatitis E infection (Deng et al., 2021; Wu et al., 2022), as well as the promising surrogate markers for quantitatively evaluating potential drug-drug interactions mediated by OATP1B (such as Rifampicin), OATP1B3 (such as micafungin), and Oatps-mediated hepatic uptake of atorvastatin (Takehara et al., 2018; Jin et al., 2022; Ma et al., 2021). The mediating effects of both oleoyl-linoleoyl-glycerol (18:1/18:2) and glycochenodeoxycholate 3-sulfate in the relationship between lipids and AAA further underscore the significance of lipid and its homeostasis in AAA. The upregulation of these three markers may serve as a risk signal for the occurrence and progression of AAA, while their downregulation could exert a protective effect. Treatment plans could consider modifying diet, exercise, or pharmacological interventions to address metabolic abnormalities associated with AAA, potentially slowing disease progression. Such recommendations would provide valuable insights for personalized treatment strategies.

This research presents numerous significant advantages. 1) By leveraging GWAS data, our MR analysis reveals novel potential causal mediators for 1,400 metabolites linked to AAAs. 2) The use of multiple cohorts derived from original GWAS data strengthens our ability to draw robust causal inferences across a large population, thereby increasing the statistical power of our findings. This makes it possible for developing potential effective drug targets and clinical trials. Nonetheless, our study has several limitations. 1) Our MR analysis is based on summary data from GWAS, while AAAs are influenced by a range of factors beyond genetic predisposition. Future research should prioritize investigating changes in relevant biomarkers to identify additional therapeutic targets for AAAs. 2) Our study predominantly involved individuals of European descent, which reduces population stratification bias but limits the generalizability of our findings to other ethnic groups. Further exploration in diverse populations is crucial to validate our results. 3) There may be overlap among participants in the GWAS cohorts, which could lead to weak instrument bias. While our F-statistics show that instrument bias is not present in the MR analysis, additional studies using independent cohorts without participant overlap are essential for deepening our understanding of the genetic factors involved in the development of AAAs.

## 5 Conclusion

Through MR and comprehensive circulating metabolomics, this study identified significant associations between metabolite expression and AAA. First, changes in the levels of specific metabolites may serve as indicators of AAA onset and progression, offering a potential strategy for the early detection of AAA. Second, certain metabolites could help predict a patient’s response to specific treatments, laying the groundwork for personalized therapy. Additionally, monitoring levels of metabolites may enable real-time assessment of treatment efficacy and guide necessary adjustments to the treatment, ultimately improving clinical outcomes and reducing side effects. By employing these strategies, personalized treatment not only enhances patient outcomes but also optimizes clinical management and overall prognosis. Therefore, the findings of this study may provide valuable insights for the clinical management of AAA and contribute to the advancement of precision medicine. These findings highlight promising avenues for the development of targeted diagnostic and therapeutic strategies for AAA, potentially improving patient outcomes and clinical management.

## Data Availability

The original contributions presented in the study are included in the article/Supplementary Material, further inquiries can be directed to the corresponding authors.
