# Supplementary material for: Assessing the relationships of 1,400 blood metabolites with abdominal aortic aneurysm: a Mendelian randomization study
Source: Front Pharmacol. 2025 Jan 3;15:1514293. doi: 10.3389/fphar.2024.1514293 (PMC11739154; doi:10.3389/fphar.2024.1514293)
Supplement: Supplementary file 1 [file DataSheet1.docx]

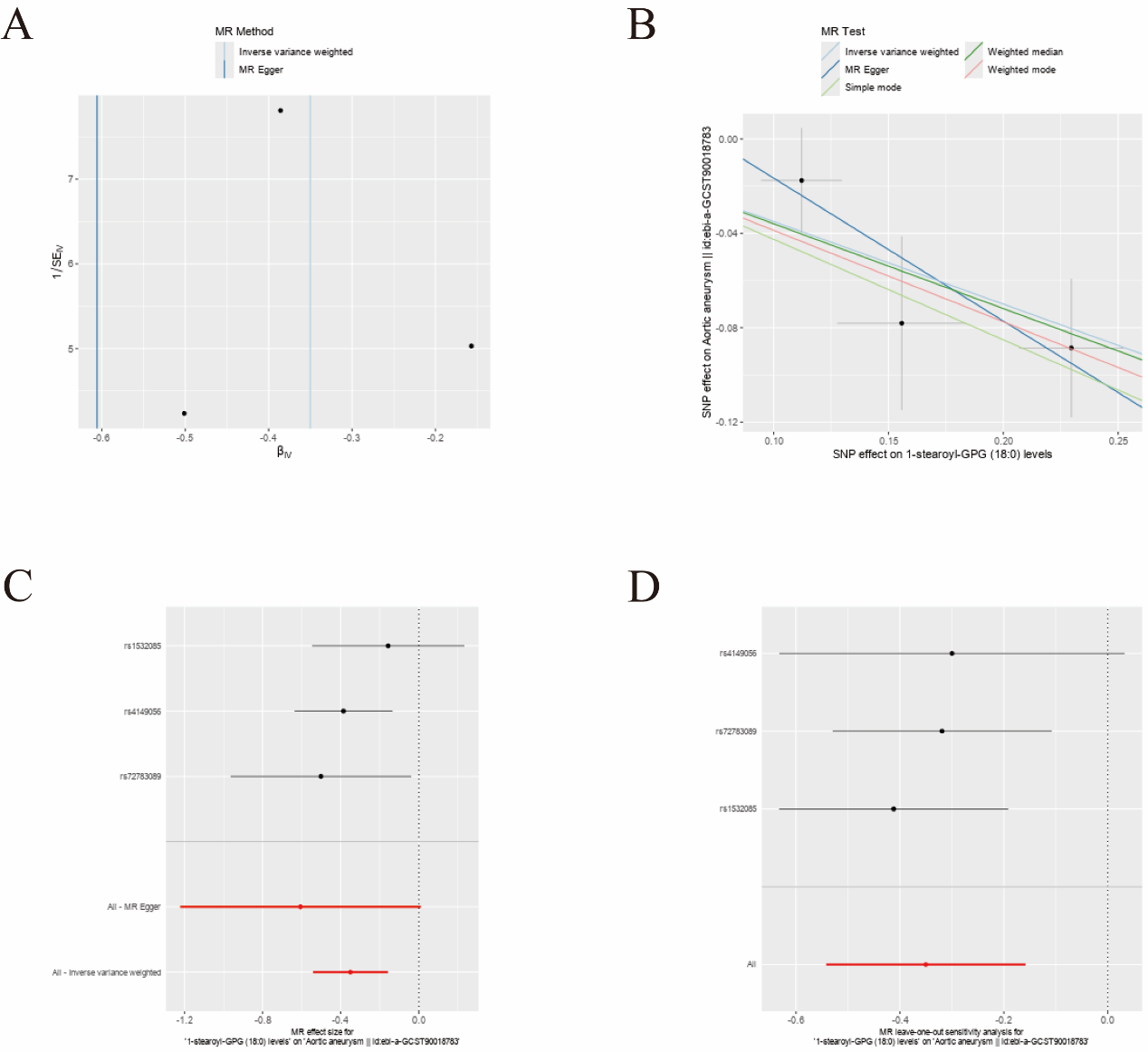


Supplementary Figure 1. The distribution of SNPs and sensitivity analyses for the causal effect of 1-stearoyl-GPG (18:0) on AAA. A, The funnel plot for the distribution of SNPs. B, Scatter plot for the causal effect. C, Forest plot of single SNP MR. D, Forest plot of leave-one-out sensitivity analysis.


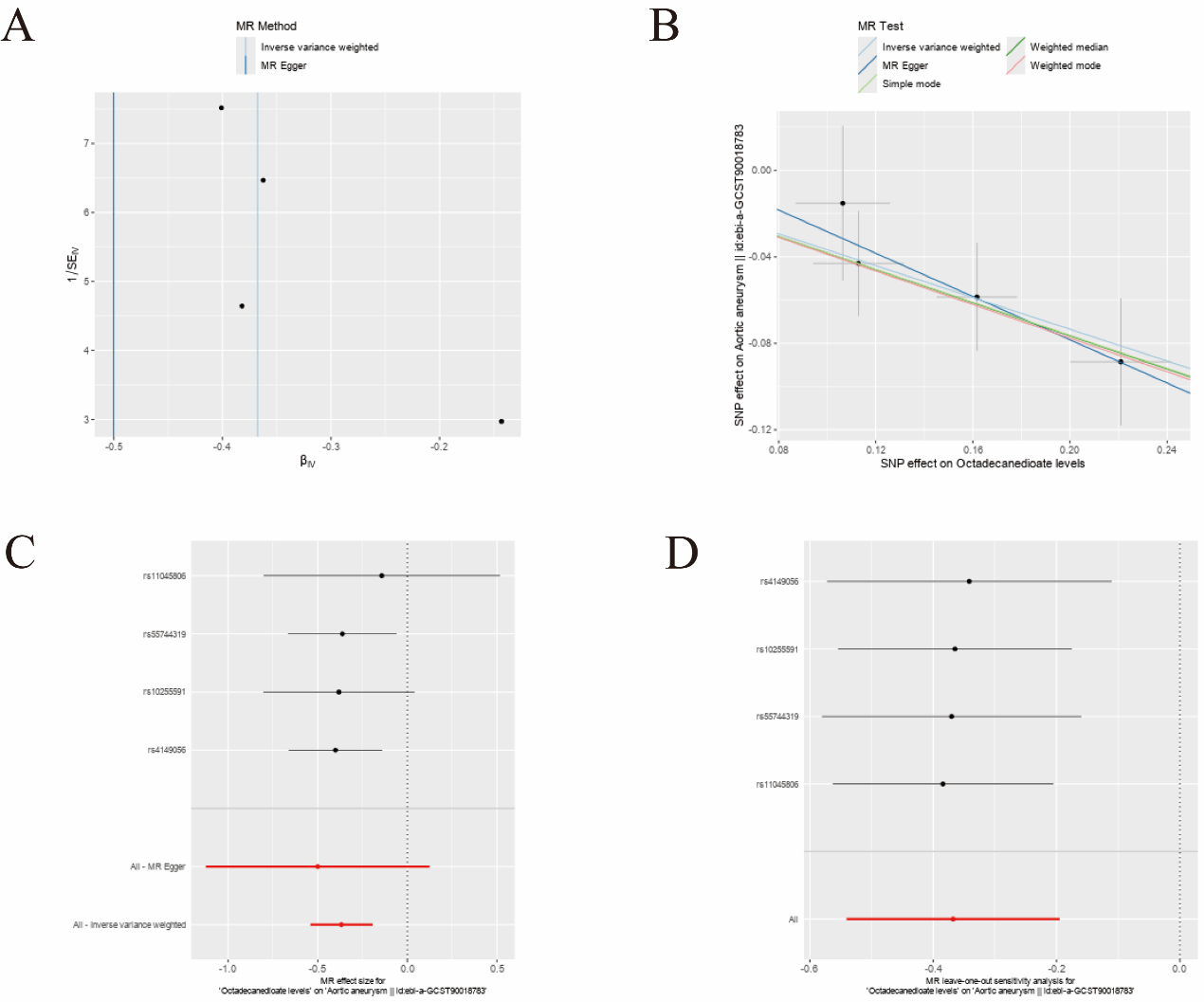


Supplementary Figure 2. The distribution of SNPs and sensitivity analyses for the causal effect of Octadecanedioate on AAA. A, The funnel plot for the distribution of SNPs. B, Scatter plot for the causal effect. C, Forest plot of single SNP MR. D, Forest plot of leave-one-out sensitivity analysis.


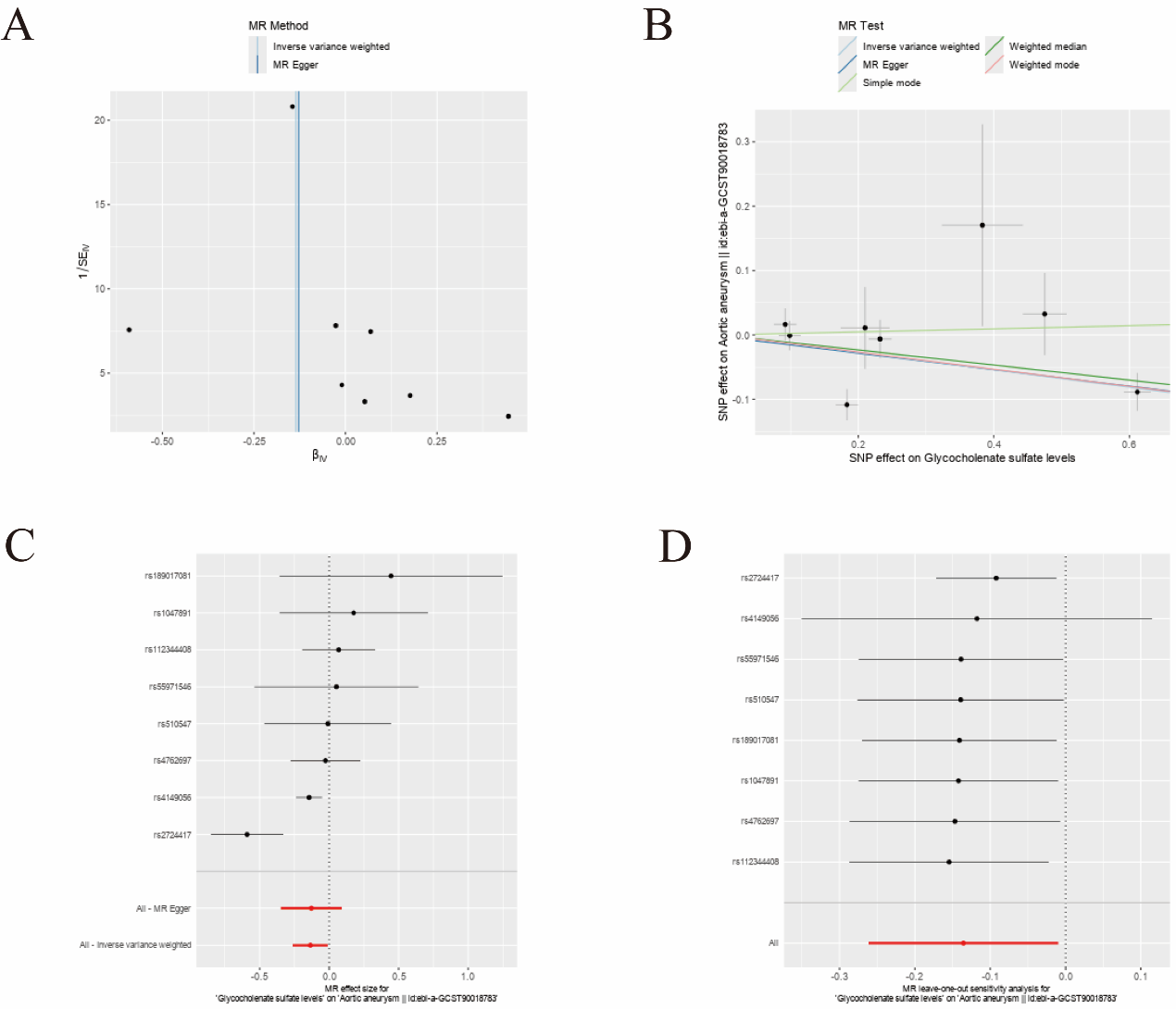


Supplementary Figure 3. The distribution of SNPs and sensitivity analyses for the causal effect of Glycocholenate sulfate on AAA. A, The funnel plot for the distribution of SNPs. B, Scatter plot for the causal effect. C, Forest plot of single SNP MR. D, Forest plot of leave-one-out sensitivity analysis.


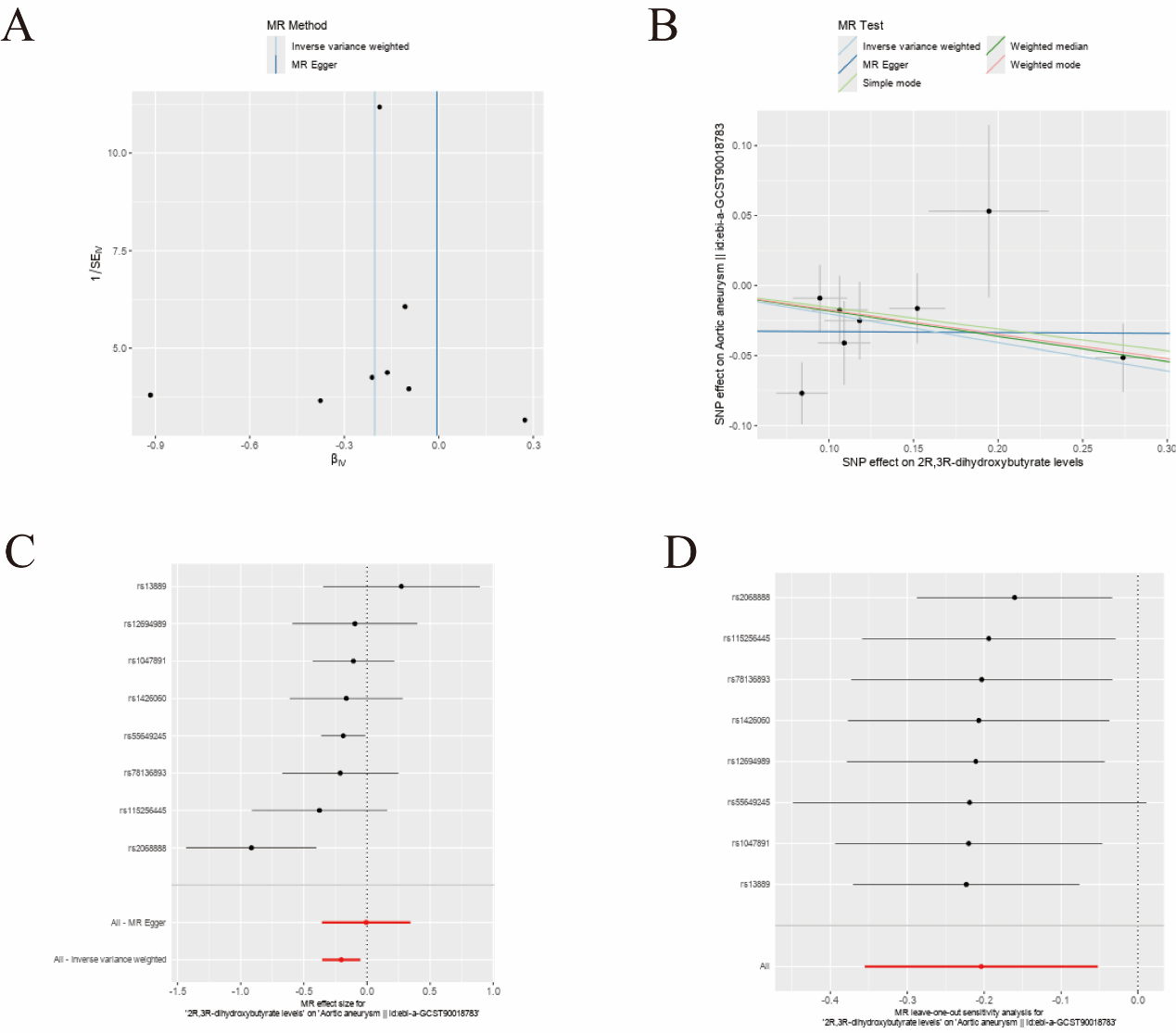


Supplementary Figure 4. The distribution of SNPs and sensitivity analyses for the causal effect of 2R,3R-dihydroxybutyrate on AAA. A, The funnel plot for the distribution of SNPs. B, Scatter plot for the causal effect. C, Forest plot of single SNP MR. D, Forest plot of leave-one-out sensitivity analysis.


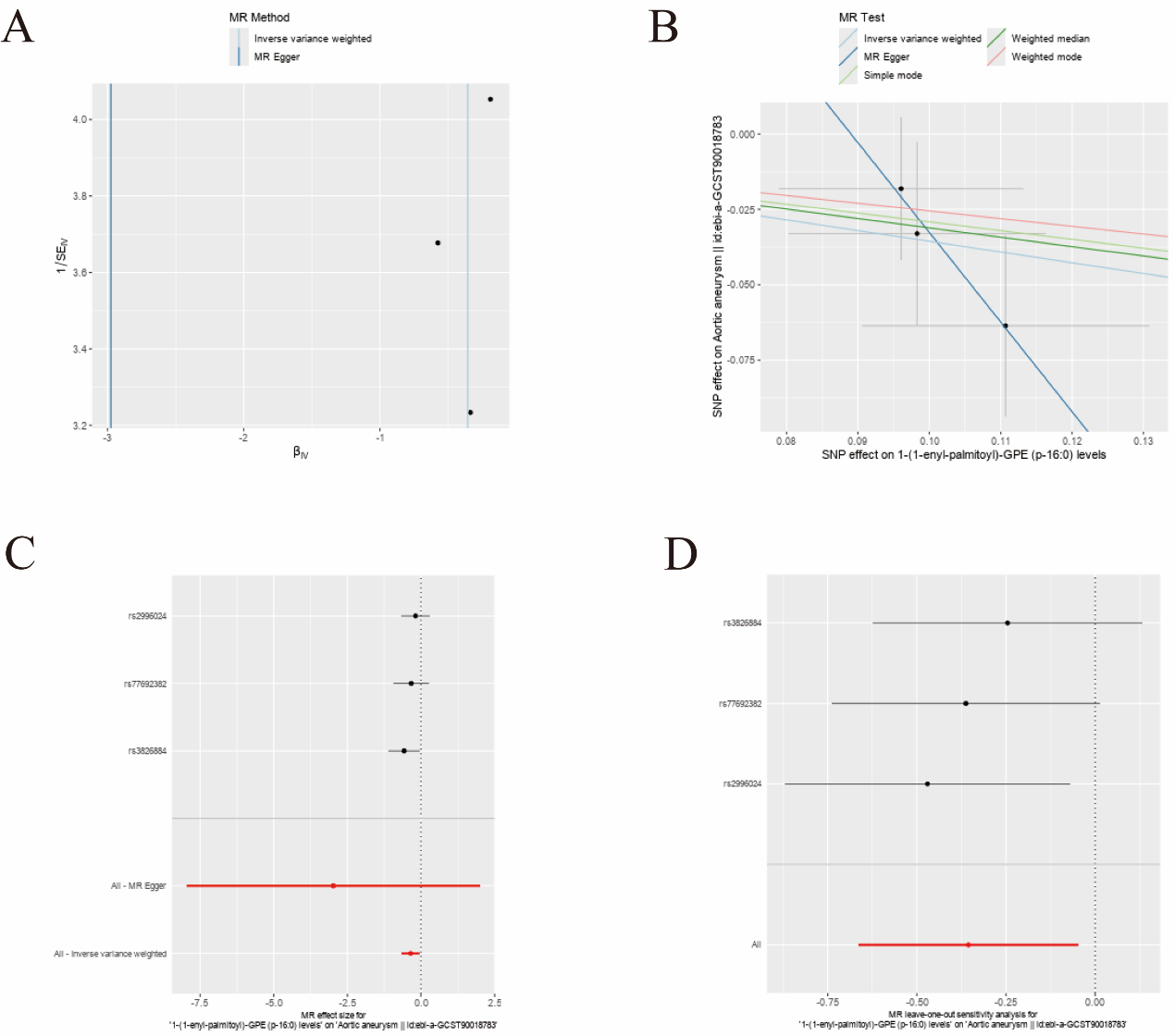


Supplementary Figure 5. The distribution of SNPs and sensitivity analyses for the causal effect of 1-(1-enyl-palmitoyl)-GPE (p-16:0) on AAA. A, The funnel plot for the distribution of SNPs. B, Scatter plot for the causal effect. C, Forest plot of single SNP MR. D, Forest plot of leave-one-out sensitivity analysis.


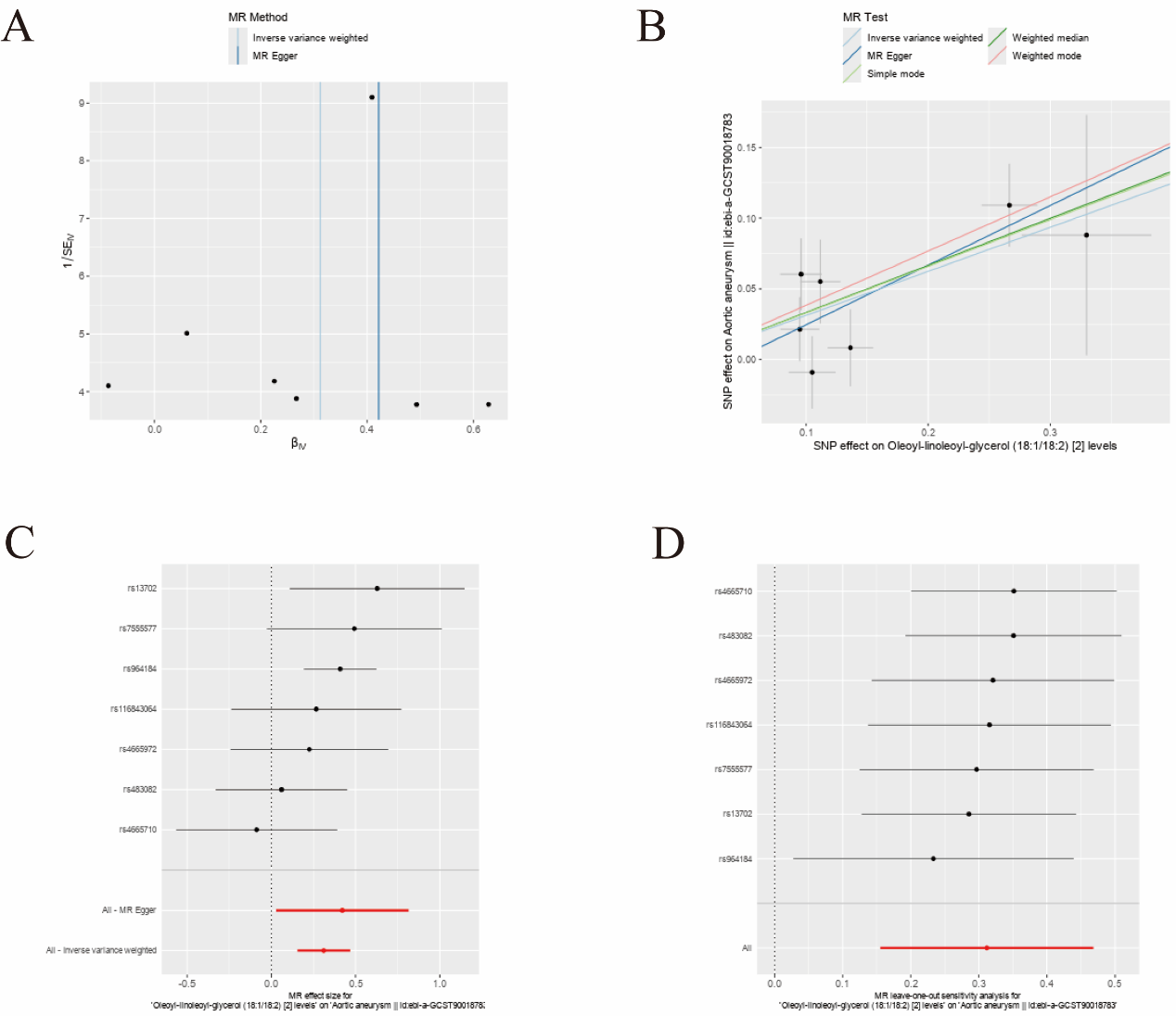


Supplementary Figure 6. The distribution of SNPs and sensitivity analyses for the causal effect of Oleoyl-linoleoyl-glycerol (18:1/18:2) [2] on AAA. A, The funnel plot for the distribution of SNPs. B, Scatter plot for the causal effect. C, Forest plot of single SNP MR. D, Forest plot of leave-one-out sensitivity analysis.


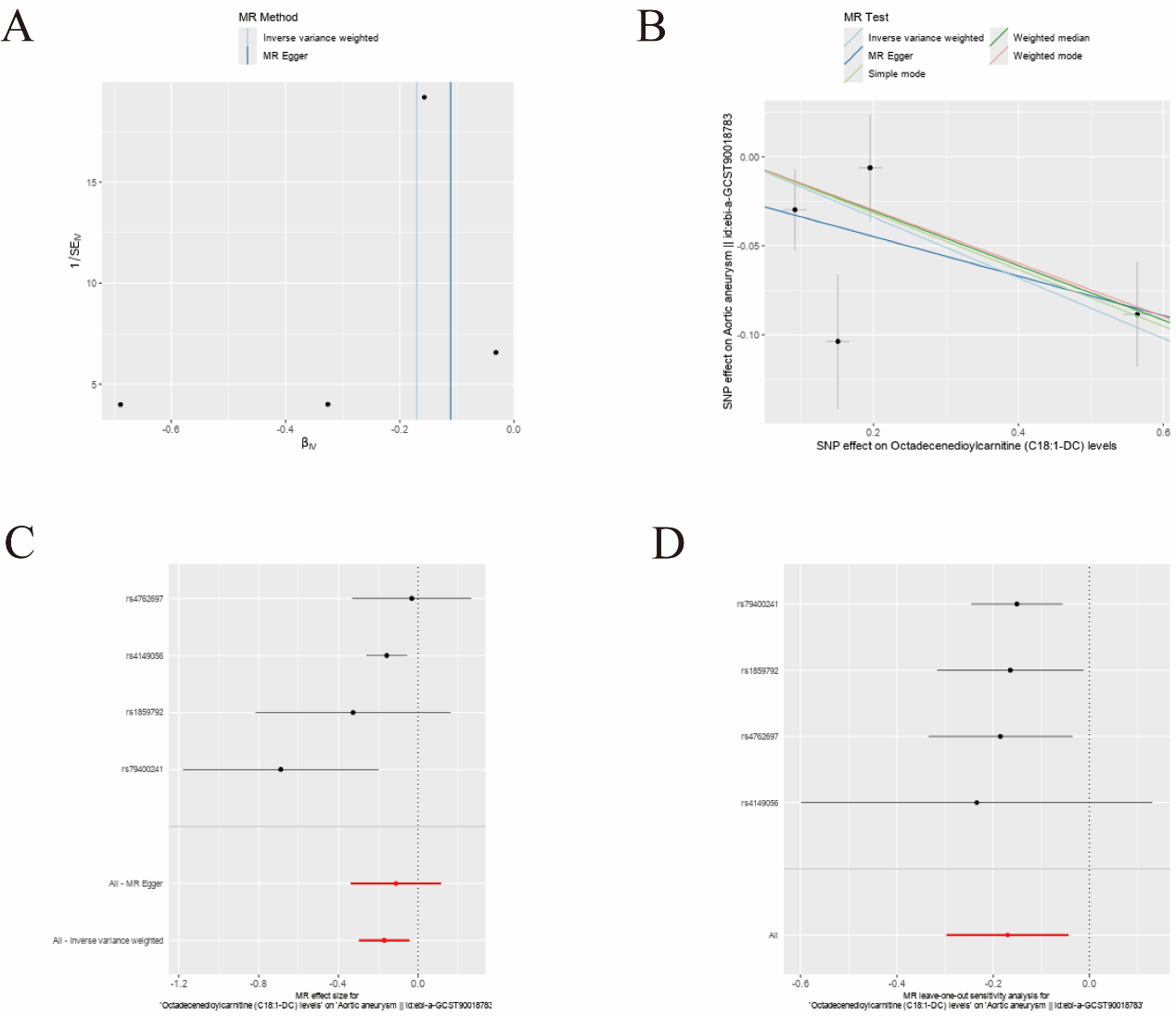


Supplementary Figure 7. The distribution of SNPs and sensitivity analyses for the causal effect of Octadecenedioylcarnitine (C18:1-DC) on AAA. A, The funnel plot for the distribution of SNPs. B, Scatter plot for the causal effect. C, Forest plot of single SNP MR. D, Forest plot of leave-one-out sensitivity analysis.


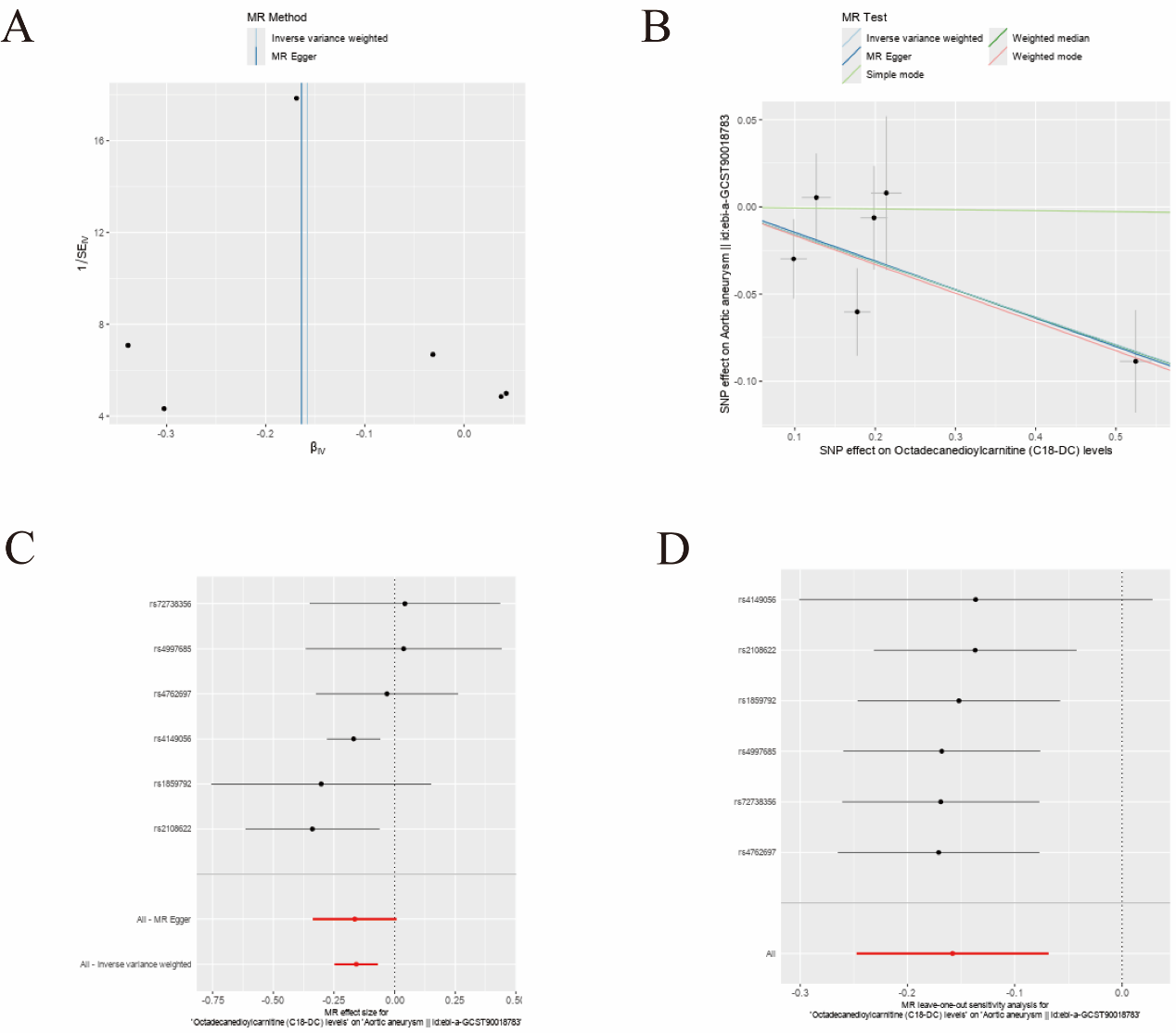


Supplementary Figure 8. The distribution of SNPs and sensitivity analyses for the causal effect of Octadecenedioylcarnitine (C18-DC) on AAA. A, The funnel plot for the distribution of SNPs. B, Scatter plot for the causal effect. C, Forest plot of single SNP MR. D, Forest plot of leave-one-out sensitivity analysis.


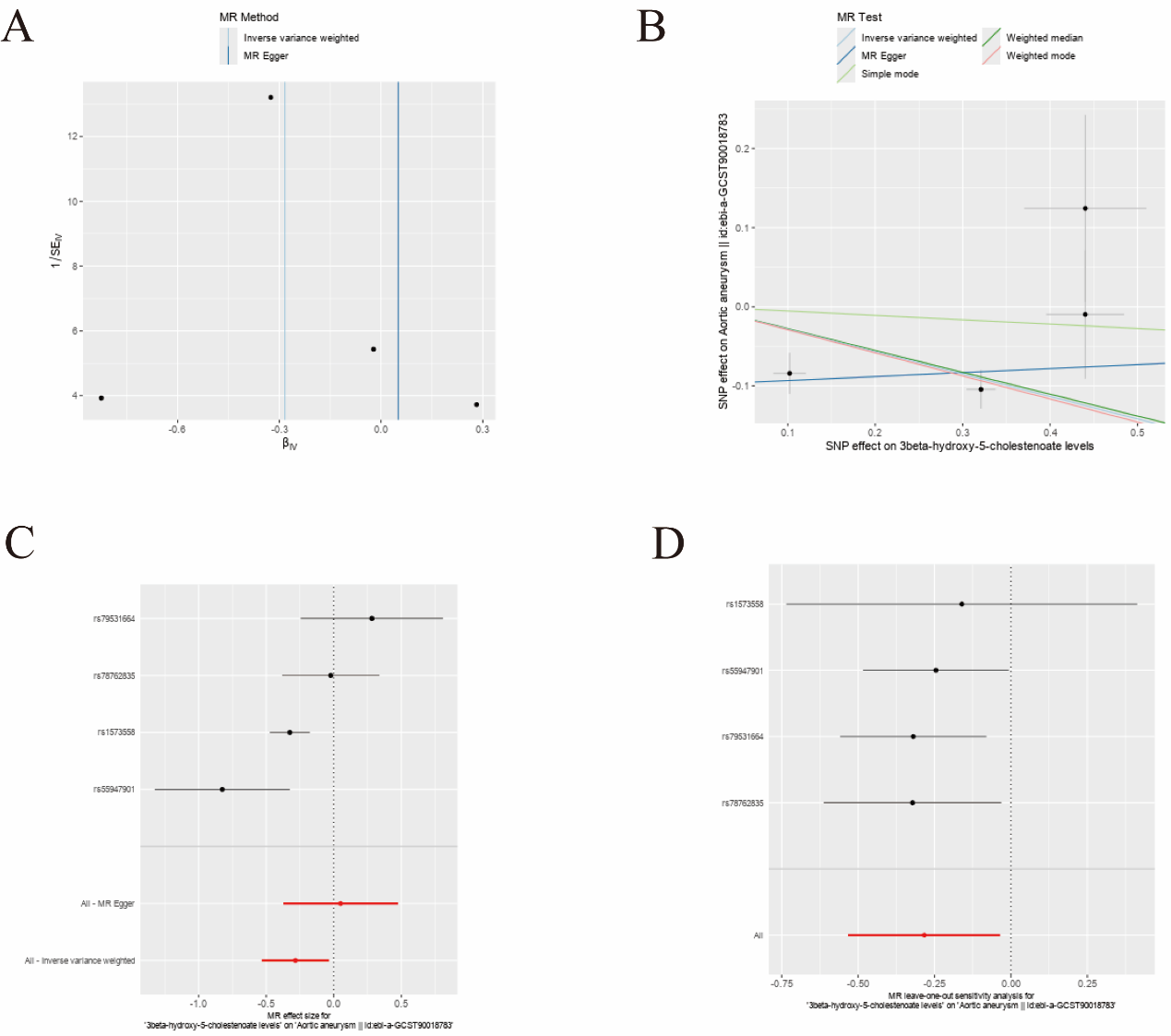


Supplementary Figure 9. The distribution of SNPs and sensitivity analyses for the causal effect of 3beta-hydroxy-5-cholestenoate on AAA. A, The funnel plot for the distribution of SNPs. B, Scatter plot for the causal effect. C, Forest plot of single SNP MR. D, Forest plot of leave-one-out sensitivity analysis.


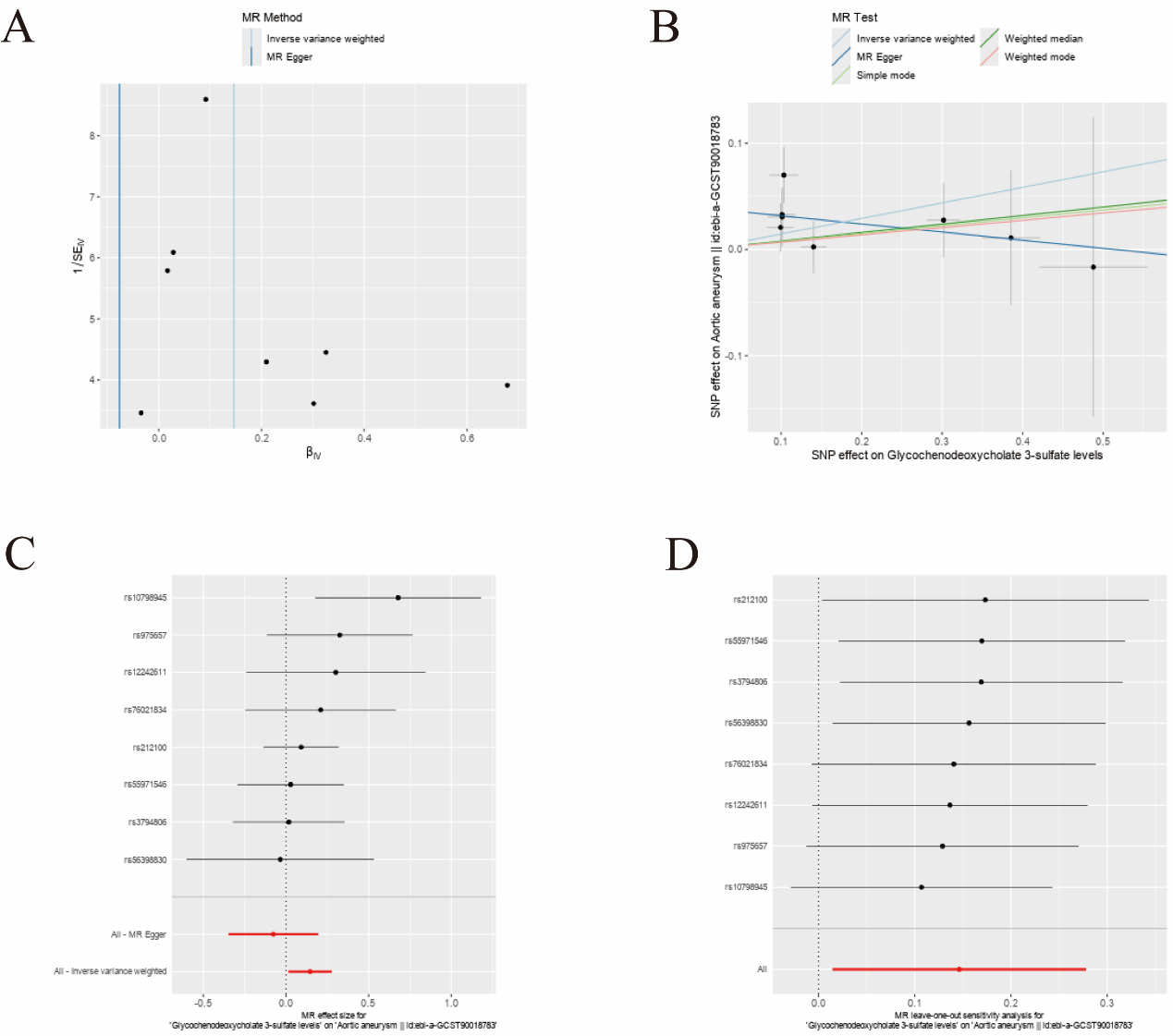


Supplementary Figure 10. The distribution of SNPs and sensitivity analyses for the causal effect of Glycochenodeoxycholate 3-sulfate on AAA. A, The funnel plot for the distribution of SNPs. B, Scatter plot for the causal effect. C, Forest plot of single SNP MR. D, Forest plot of leave-one-out sensitivity analysis.


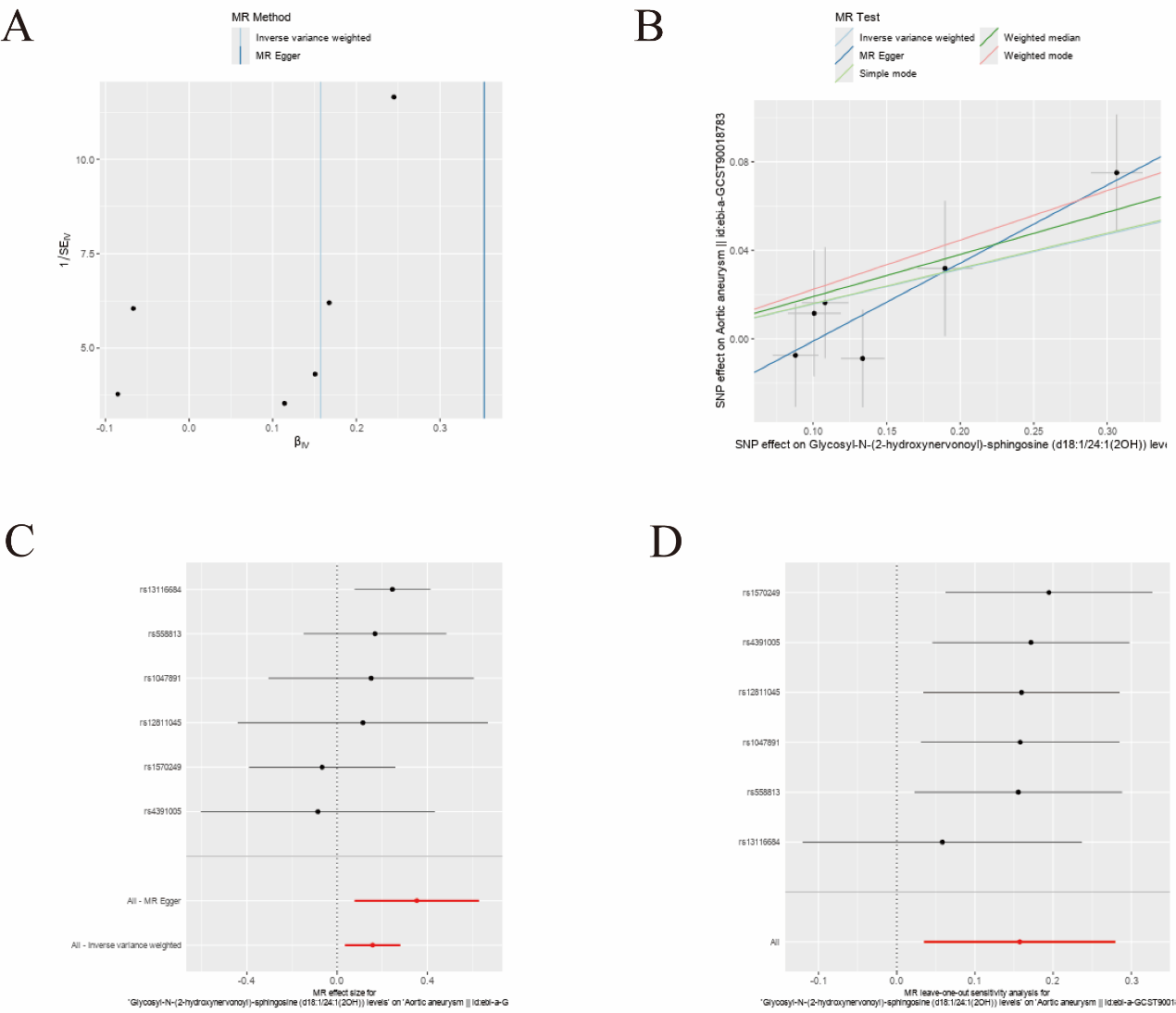


Supplementary Figure 11. The distribution of SNPs and sensitivity analyses for the causal effect of Glycosyl-N-(2-hydroxynervonoyl)-sphingosine (d18:1/24:1(2OH)) on AAA. A, The funnel plot for the distribution of SNPs. B, Scatter plot for the causal effect. C, Forest plot of single SNP MR. D, Forest plot of leave-one-out sensitivity analysis.


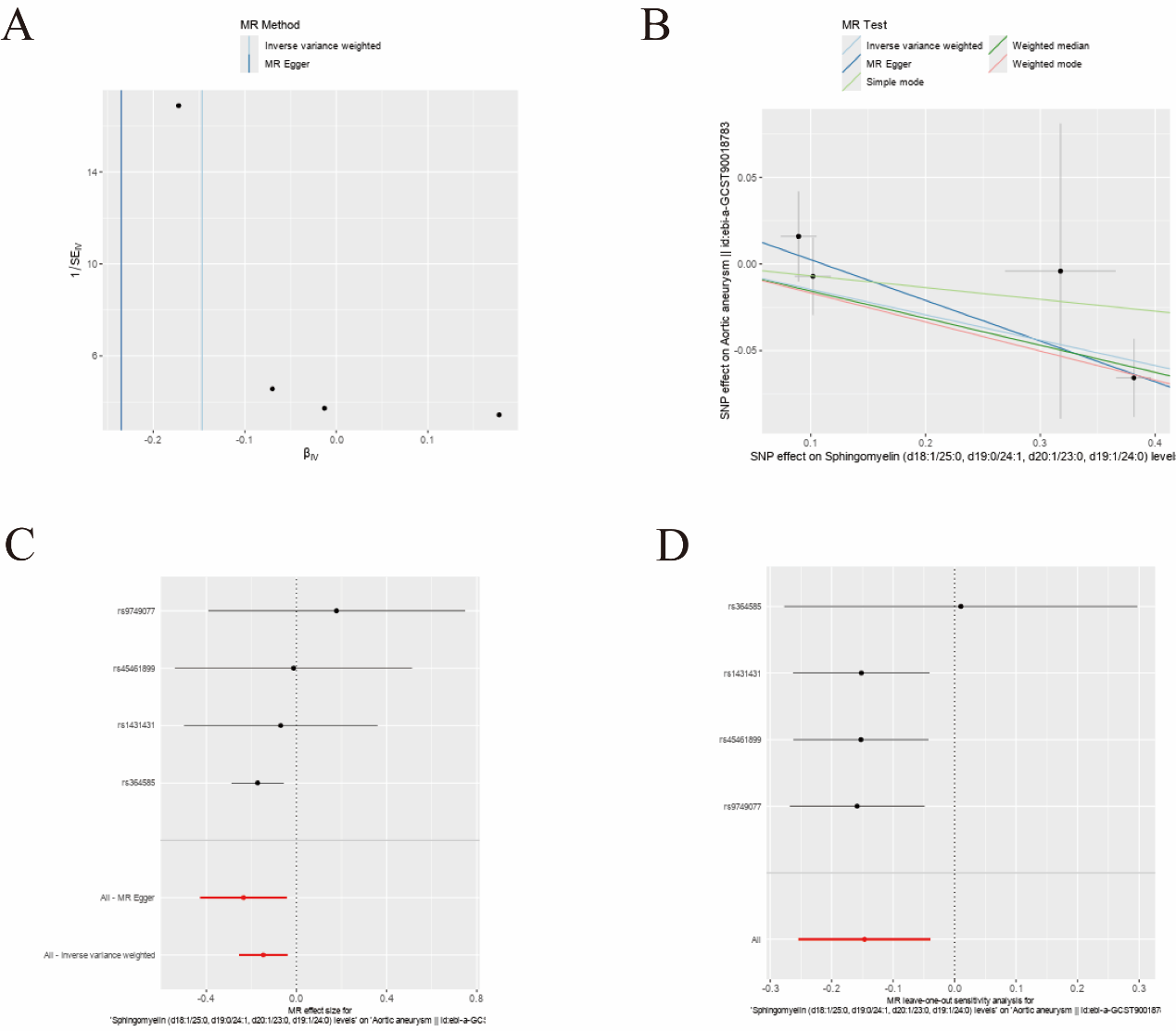


Supplementary Figure 12. The distribution of SNPs and sensitivity analyses for the causal effect of Sphingomyelin (d18:1/25:0, d19:0/24:1, d20:1/23:0, d19:1/24:0) on AAA. A, The funnel plot for the distribution of SNPs. B, Scatter plot for the causal effect. C, Forest plot of single SNP MR. D, Forest plot of leave-one-out sensitivity analysis.


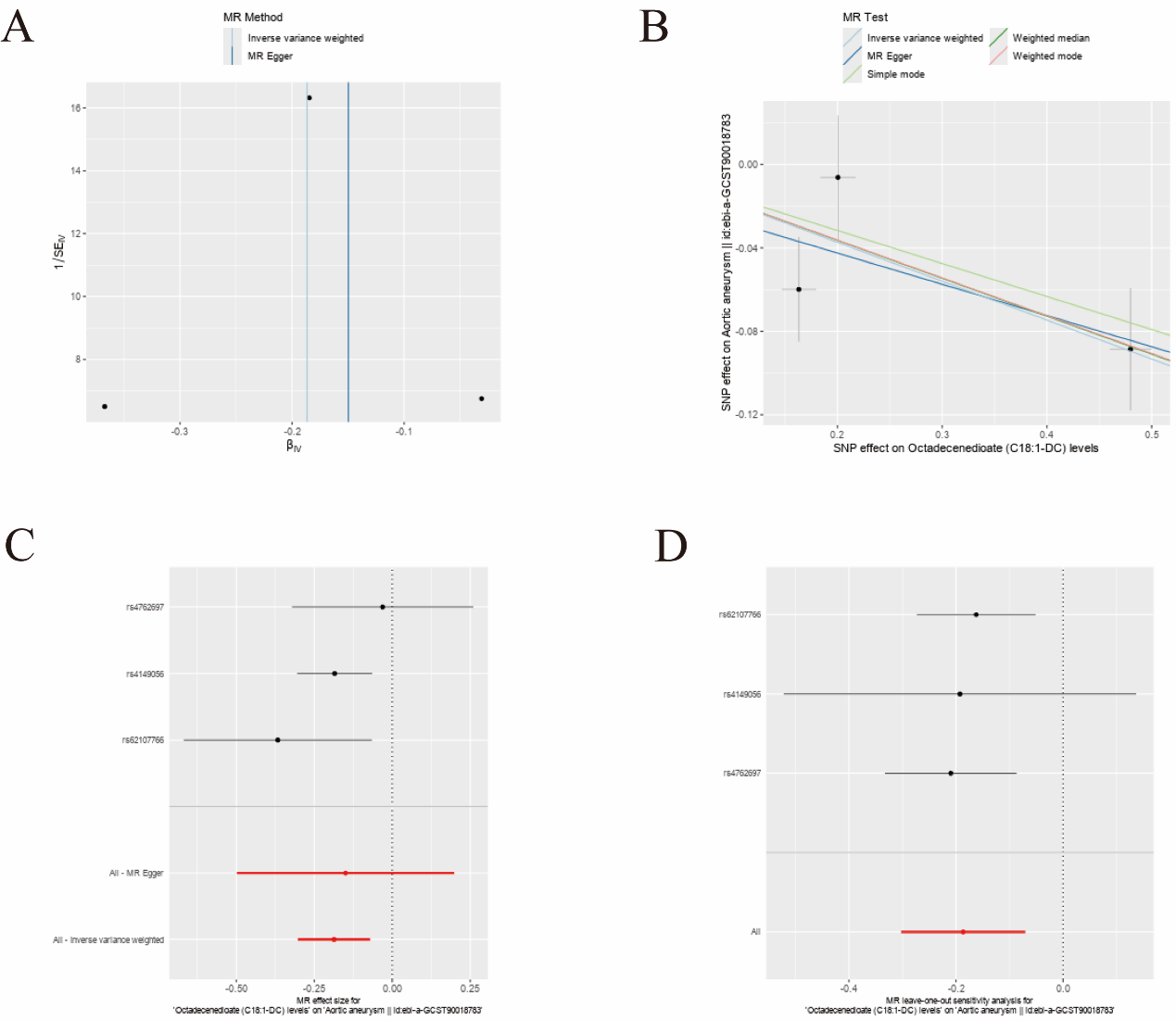


Supplementary Figure 13. The distribution of SNPs and sensitivity analyses for the causal effect of Octadecenedioate (C18:1-DC) on AAA. A, The funnel plot for the distribution of SNPs. B, Scatter plot for the causal effect. C, Forest plot of single SNP MR. D, Forest plot of leave-one-out sensitivity analysis.


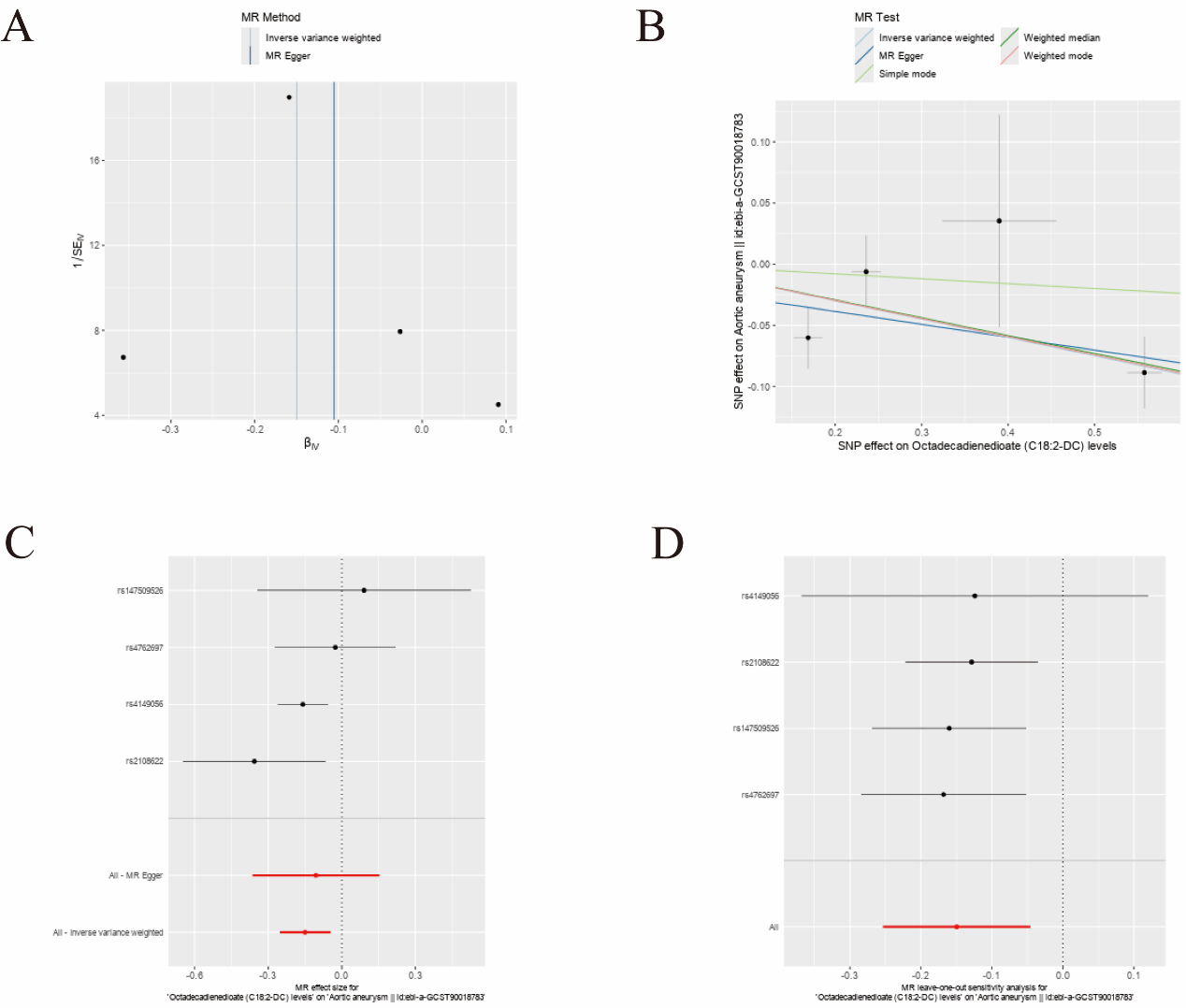


Supplementary Figure 14. The distribution of SNPs and sensitivity analyses for the causal effect of Octadecadienedioate (C18:2-DC) on AAA. A, The funnel plot for the distribution of SNPs. B, Scatter plot for the causal effect. C, Forest plot of single SNP MR. D, Forest plot of leave-one-out sensitivity analysis.


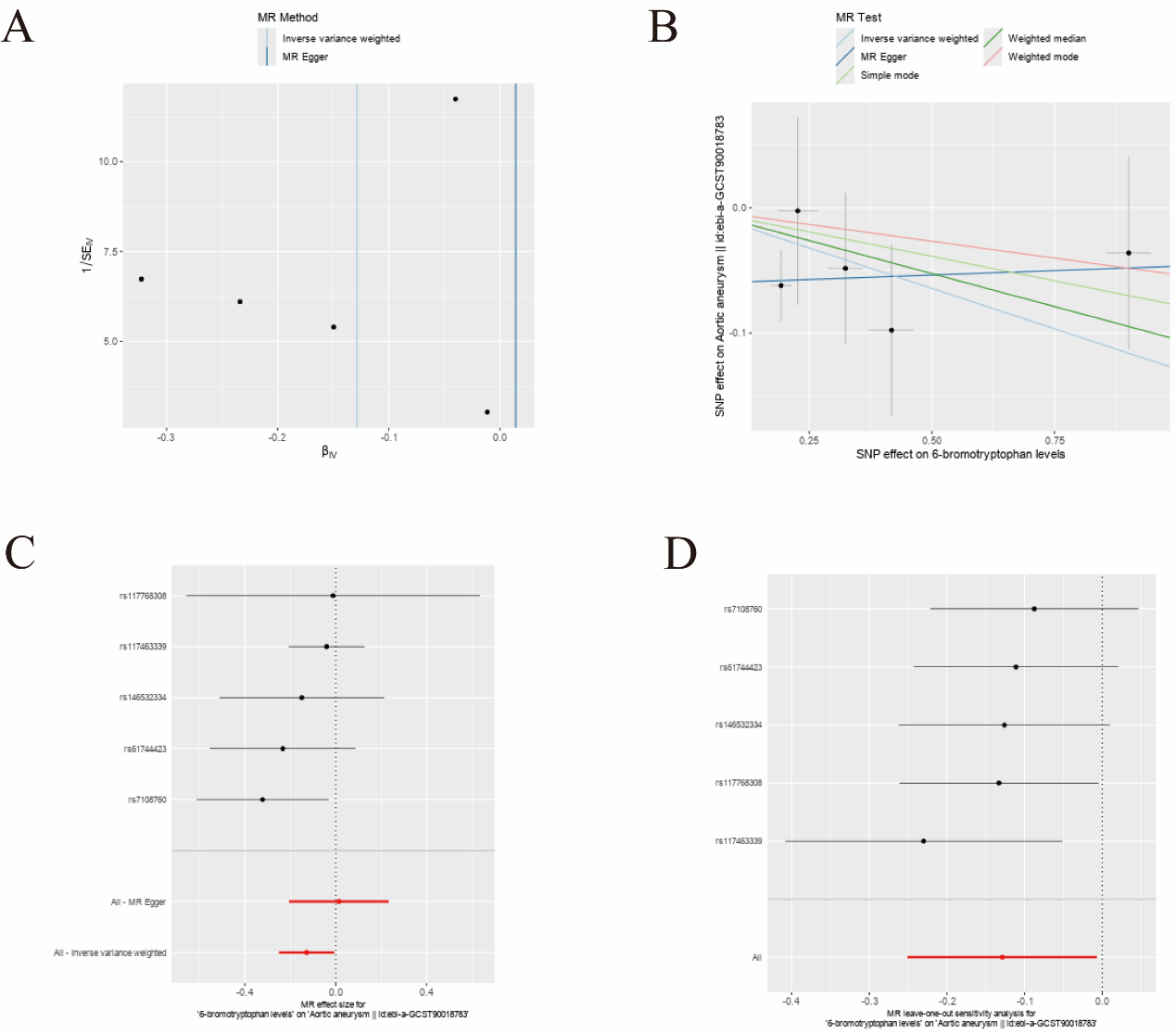


Supplementary Figure 15. The distribution of SNPs and sensitivity analyses for the causal effect of 6-bromotryptophan on AAA. A, The funnel plot for the distribution of SNPs. B, Scatter plot for the causal effect. C, Forest plot of single SNP MR. D, Forest plot of leave-one-out sensitivity analysis.


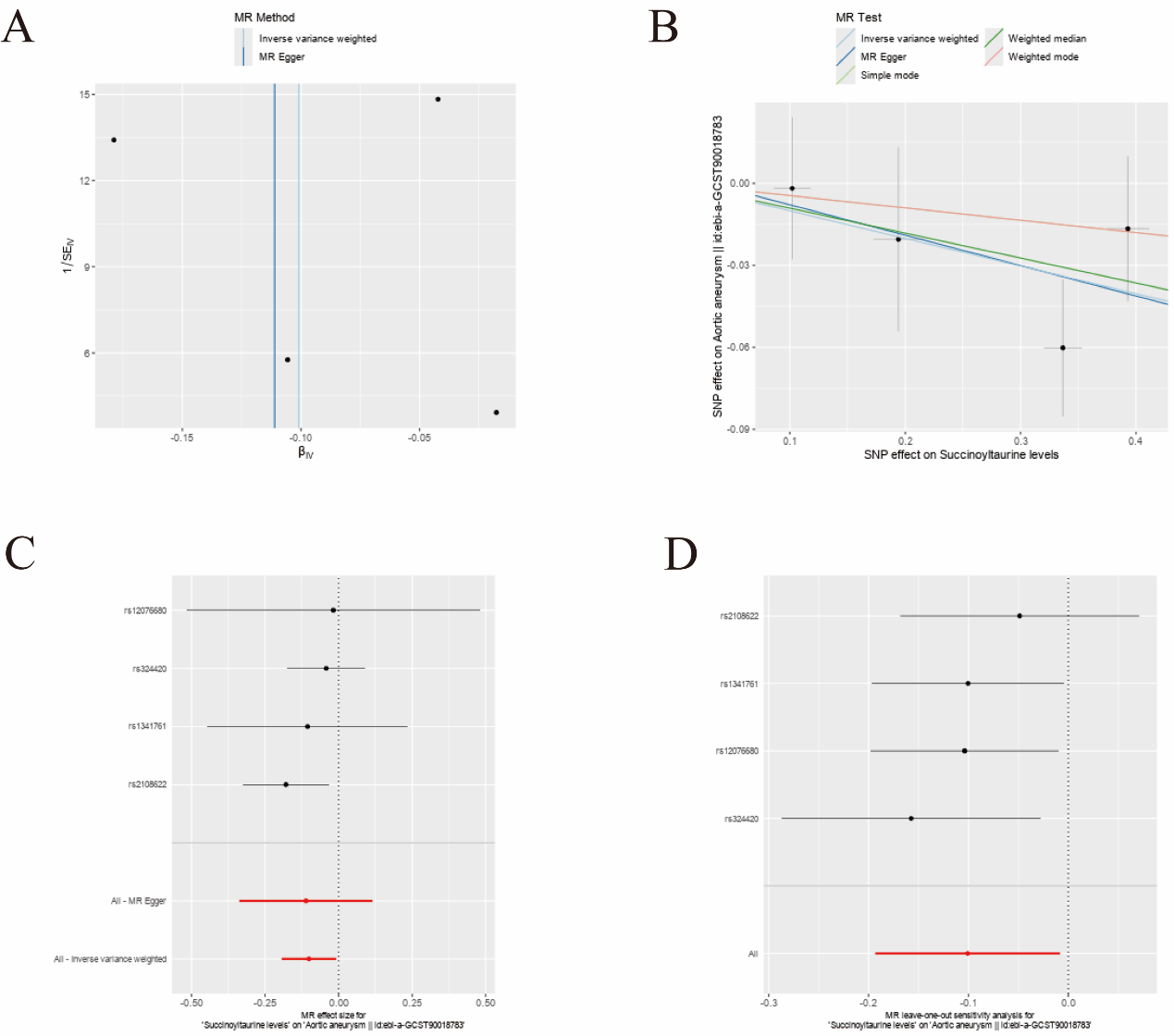


Supplementary Figure 16. The distribution of SNPs and sensitivity analyses for the causal effect of Succinoyltaurine on AAA. A, The funnel plot for the distribution of SNPs. B, Scatter plot for the causal effect. C, Forest plot of single SNP MR. D, Forest plot of leave-one-out sensitivity analysis.


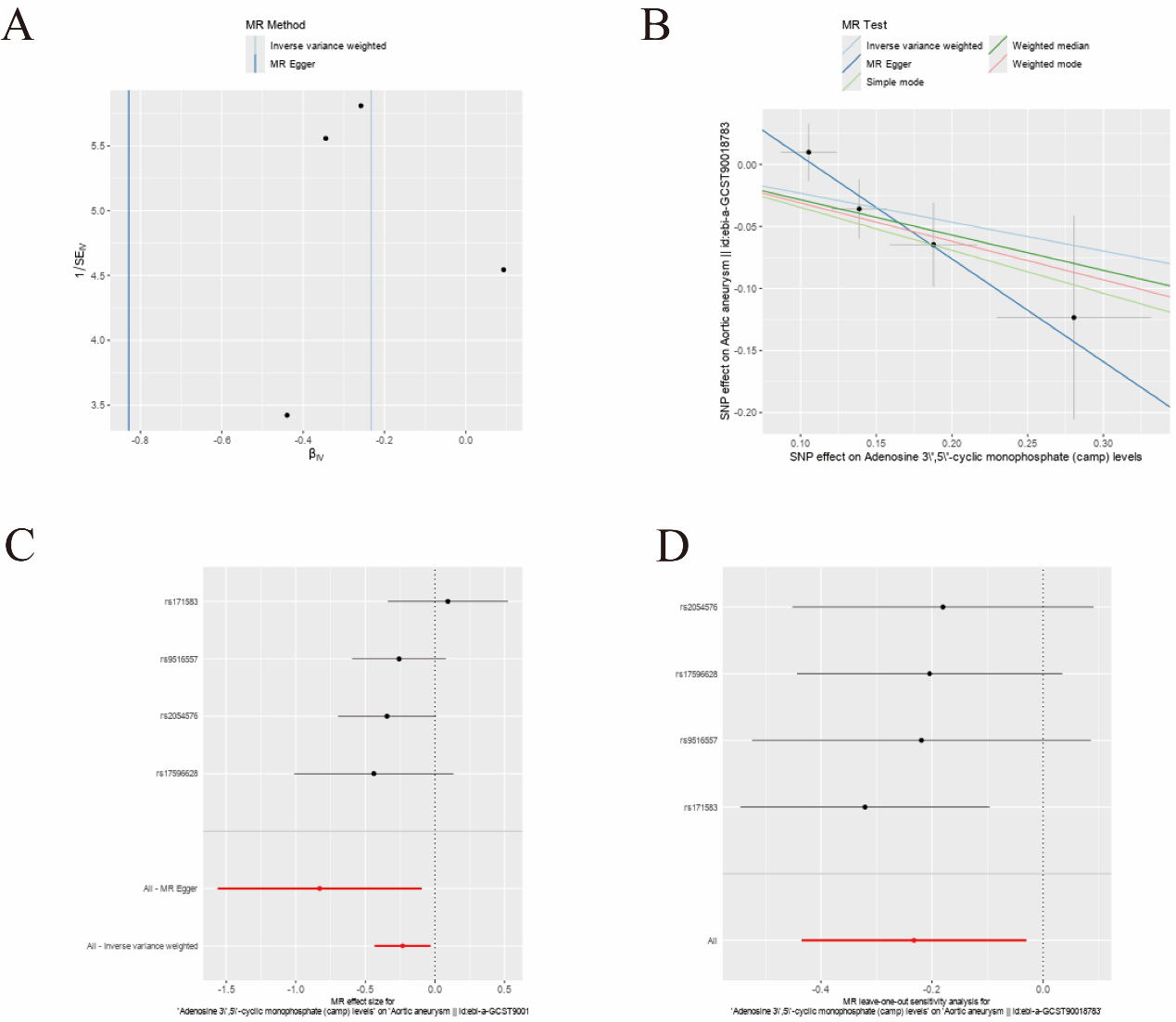


Supplementary Figure 17. The distribution of SNPs and sensitivity analyses for the causal effect of Adenosine 3\’,5\’-cyclic monophosphate(camp) on AAA. A, The funnel plot for the distribution of SNPs. B, Scatter plot for the causal effect. C, Forest plot of single SNP MR. D, Forest plot of leave-one-out sensitivity analysis.


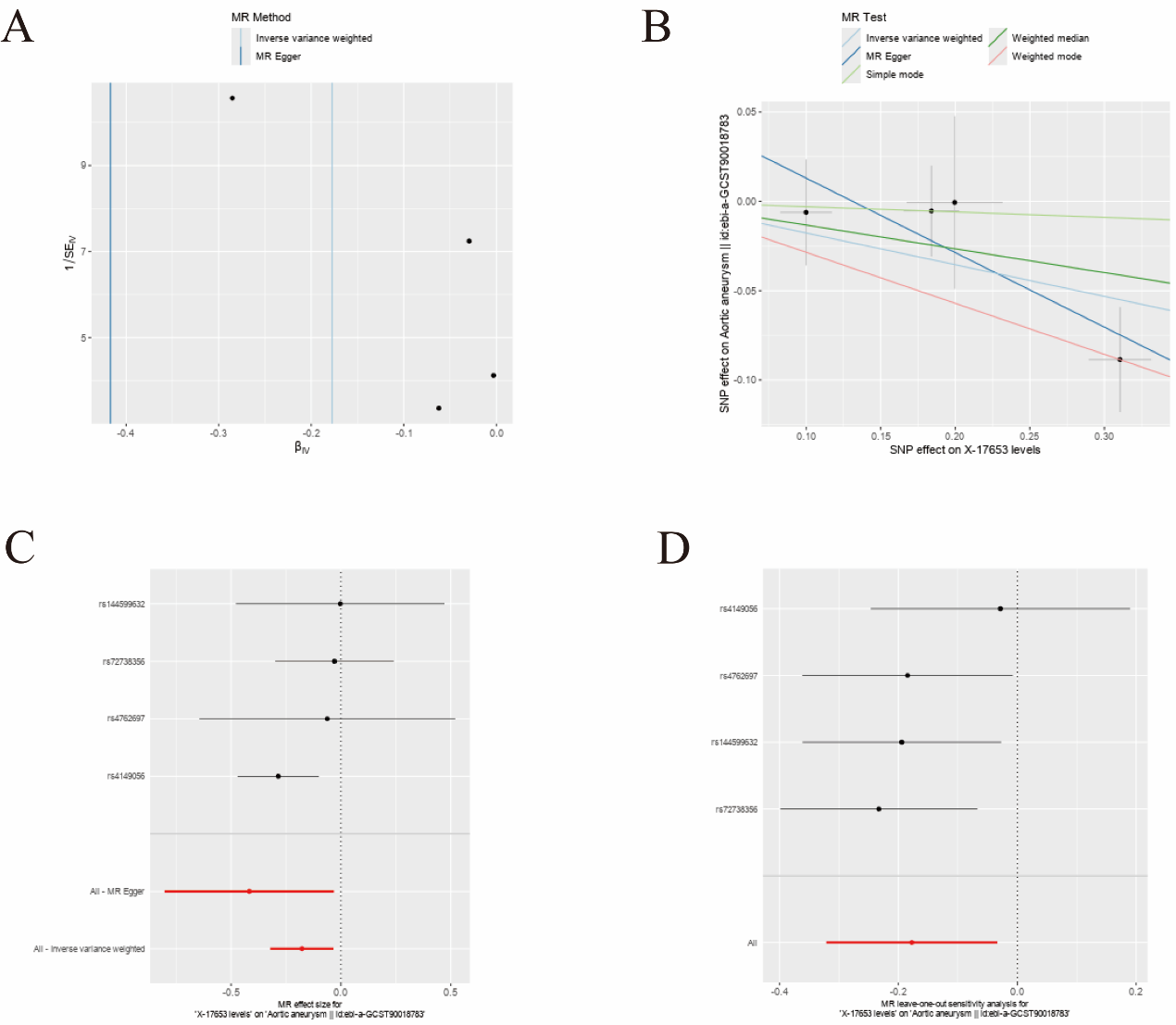


Supplementary Figure 18. The distribution of SNPs and sensitivity analyses for the causal effect of X-17653 on AAA. A, The funnel plot for the distribution of SNPs. B, Scatter plot for the causal effect. C, Forest plot of single SNP MR. D, Forest plot of leave-one-out sensitivity analysis.


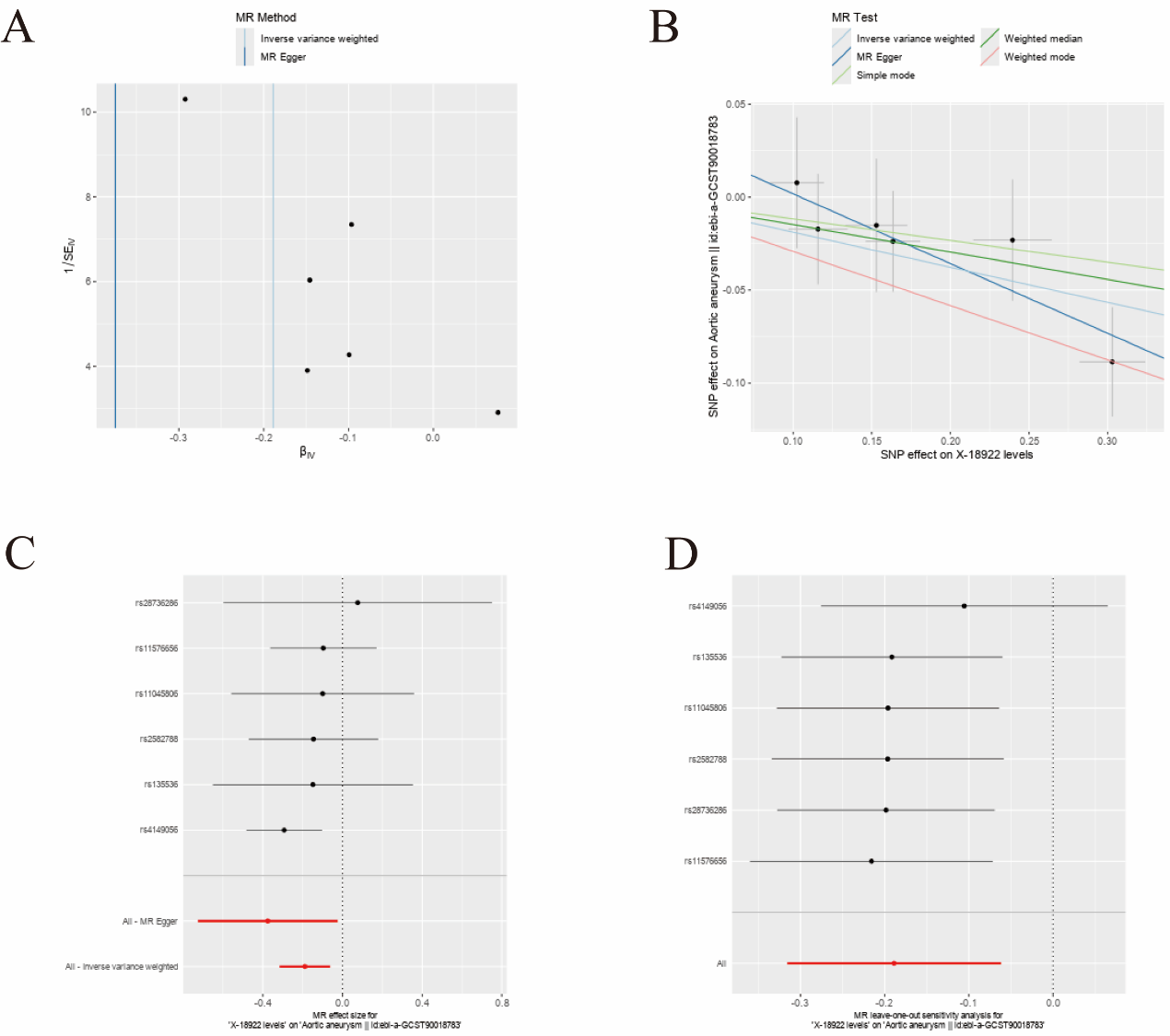


Supplementary Figure 19. The distribution of SNPs and sensitivity analyses for the causal effect of X-18922 on AAA. A, The funnel plot for the distribution of SNPs. B, Scatter plot for the causal effect. C, Forest plot of single SNP MR. D, Forest plot of leave-one-out sensitivity analysis.


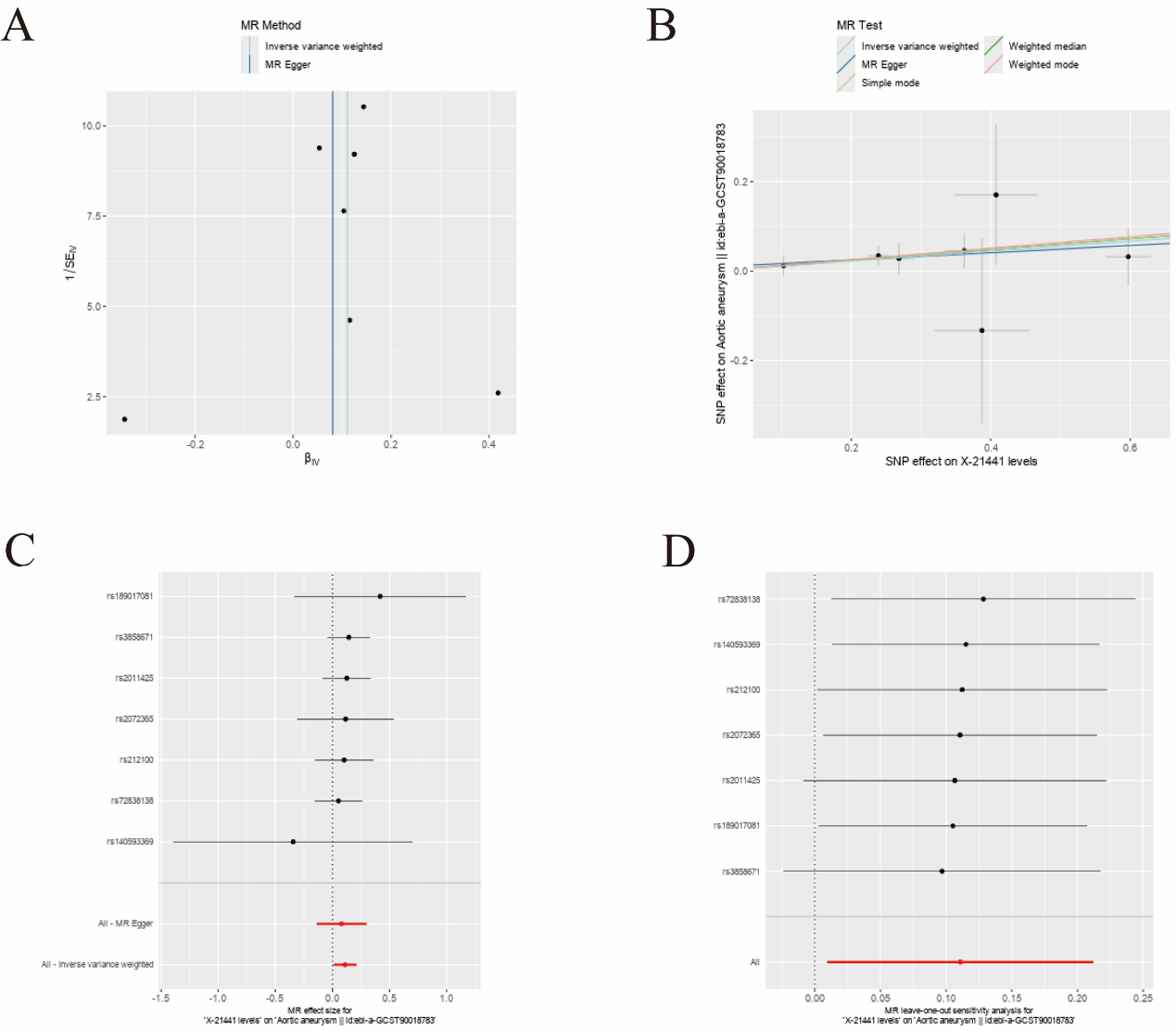


Supplementary Figure 20. The distribution of SNPs and sensitivity analyses for the causal effect of X-21441 on AAA. A, The funnel plot for the distribution of SNPs. B, Scatter plot for the causal effect. C, Forest plot of single SNP MR. D, Forest plot of leave-one-out sensitivity analysis.


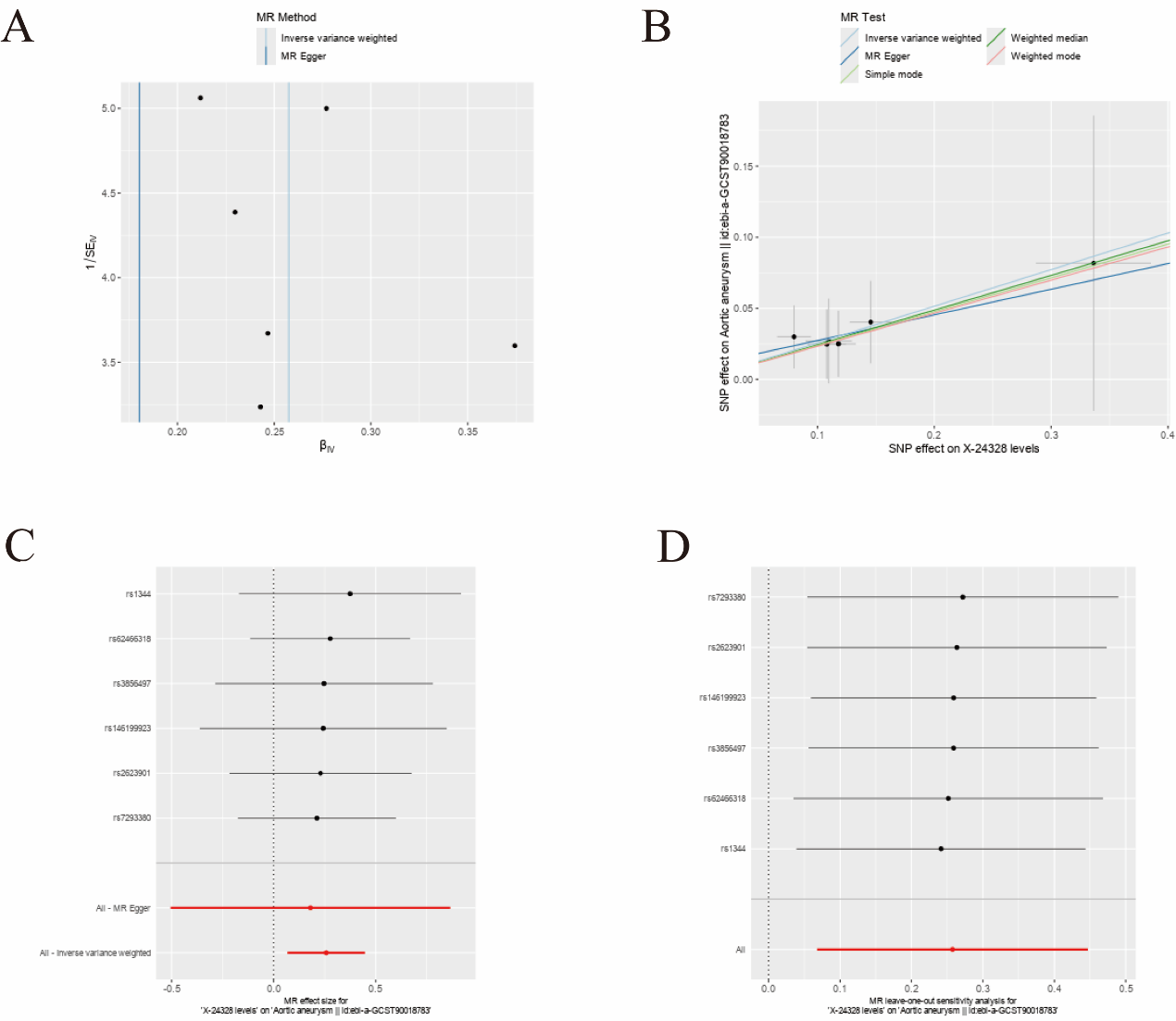


Supplementary Figure 21. The distribution of SNPs and sensitivity analyses for the causal effect of X-24328 on AAA. A, The funnel plot for the distribution of SNPs. B, Scatter plot for the causal effect. C, Forest plot of single SNP MR. D, Forest plot of leave-one-out sensitivity analysis.


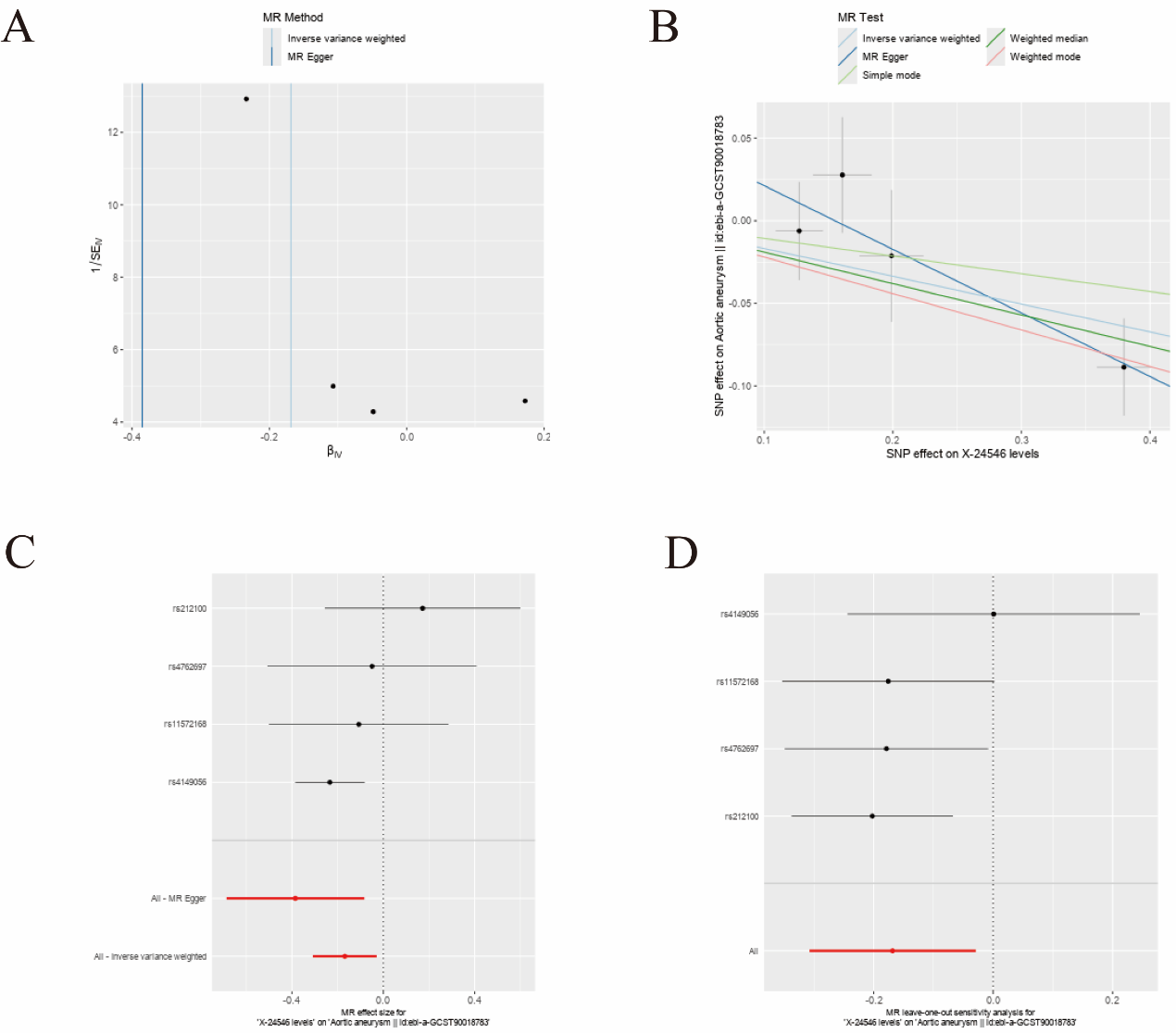


Supplementary Figure 22. The distribution of SNPs and sensitivity analyses for the causal effect of X-24546 on AAA. A, The funnel plot for the distribution of SNPs. B, Scatter plot for the causal effect. C, Forest plot of single SNP MR. D, Forest plot of leave-one-out sensitivity analysis.


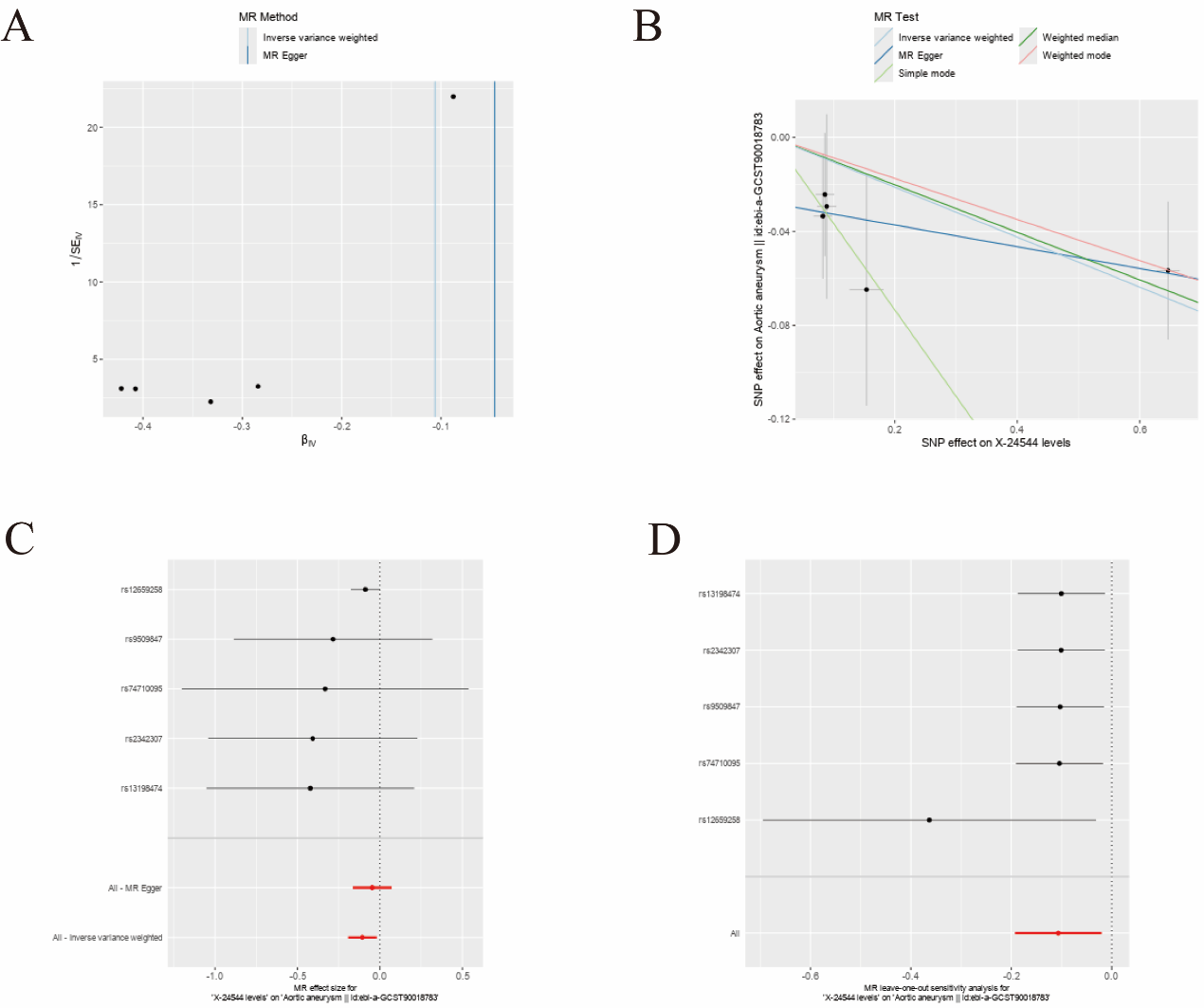


Supplementary Figure 23. The distribution of SNPs and sensitivity analyses for the causal effect of X-24544 on AAA. A, The funnel plot for the distribution of SNPs. B, Scatter plot for the causal effect. C, Forest plot of single SNP MR. D, Forest plot of leave-one-out sensitivity analysis.


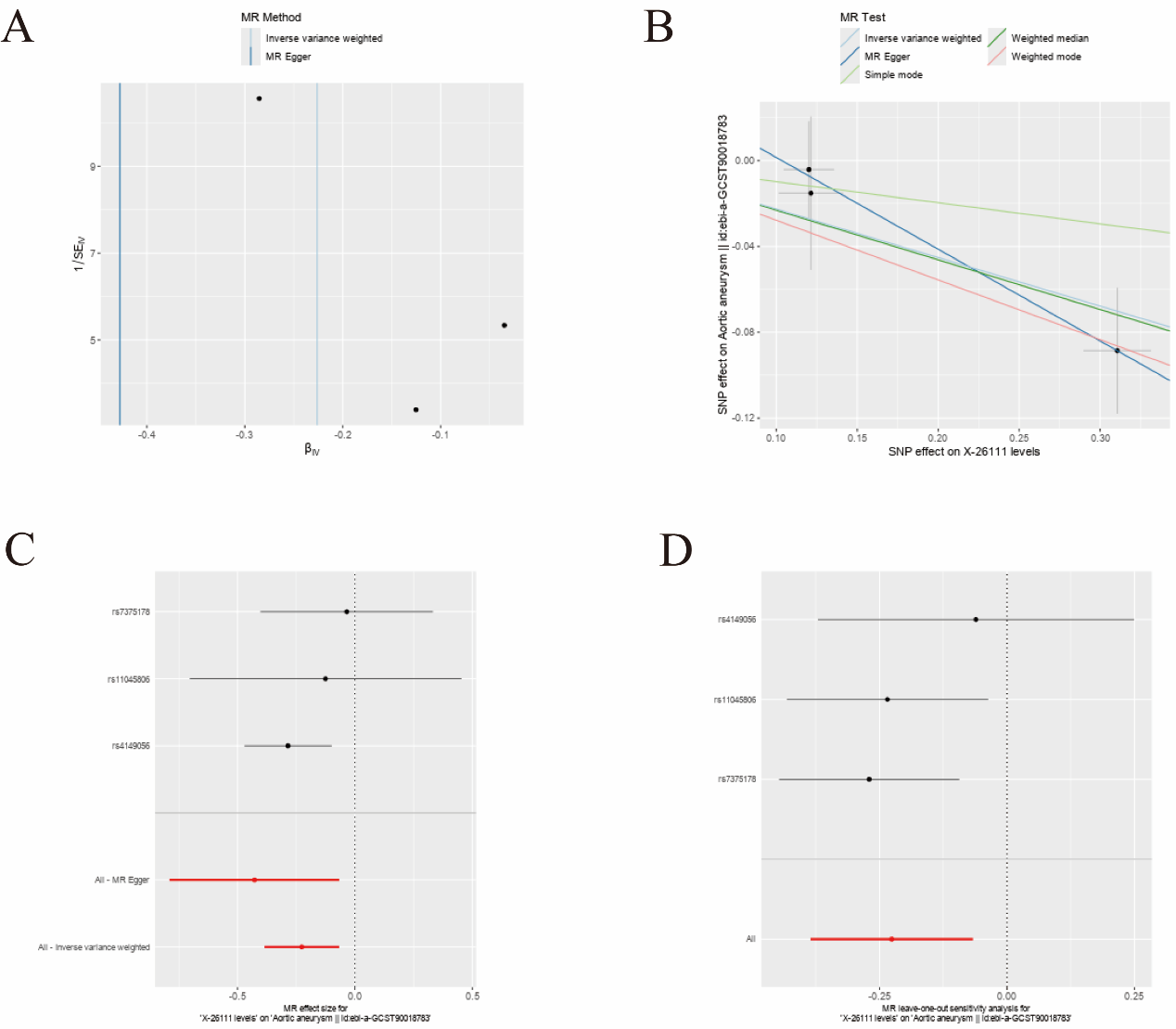


Supplementary Figure 24. The distribution of SNPs and sensitivity analyses for the causal effect of X-26111 on AAA. A, The funnel plot for the distribution of SNPs. B, Scatter plot for the causal effect. C, Forest plot of single SNP MR. D, Forest plot of leave-one-out sensitivity analysis.


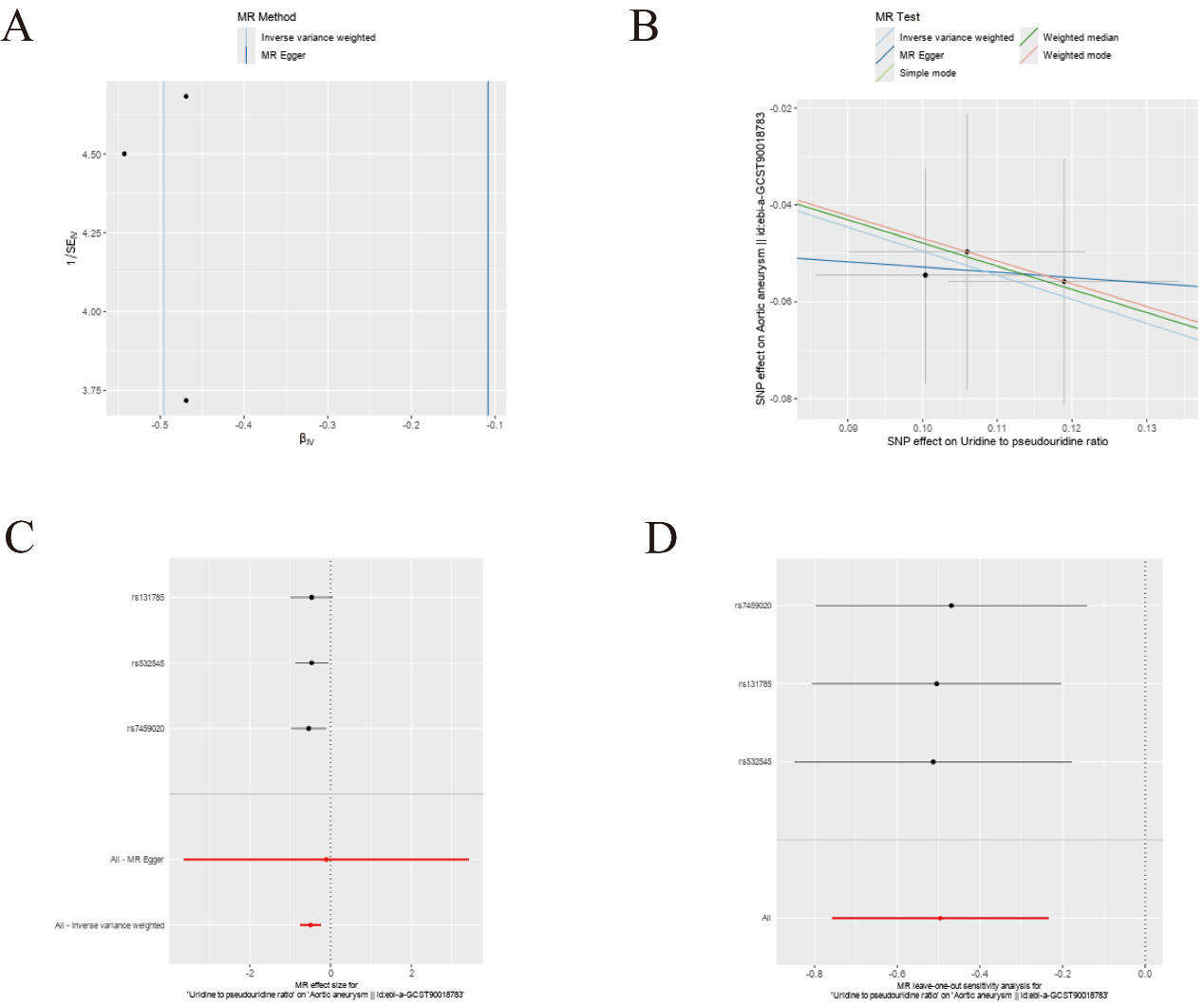


Supplementary Figure 25. The distribution of SNPs and sensitivity analyses for the causal effect of Uridine to pseudouridine ratio on AAA. A, The funnel plot for the distribution of SNPs. B, Scatter plot for the causal effect. C, Forest plot of single SNP MR. D, Forest plot of leave-one-out sensitivity analysis.


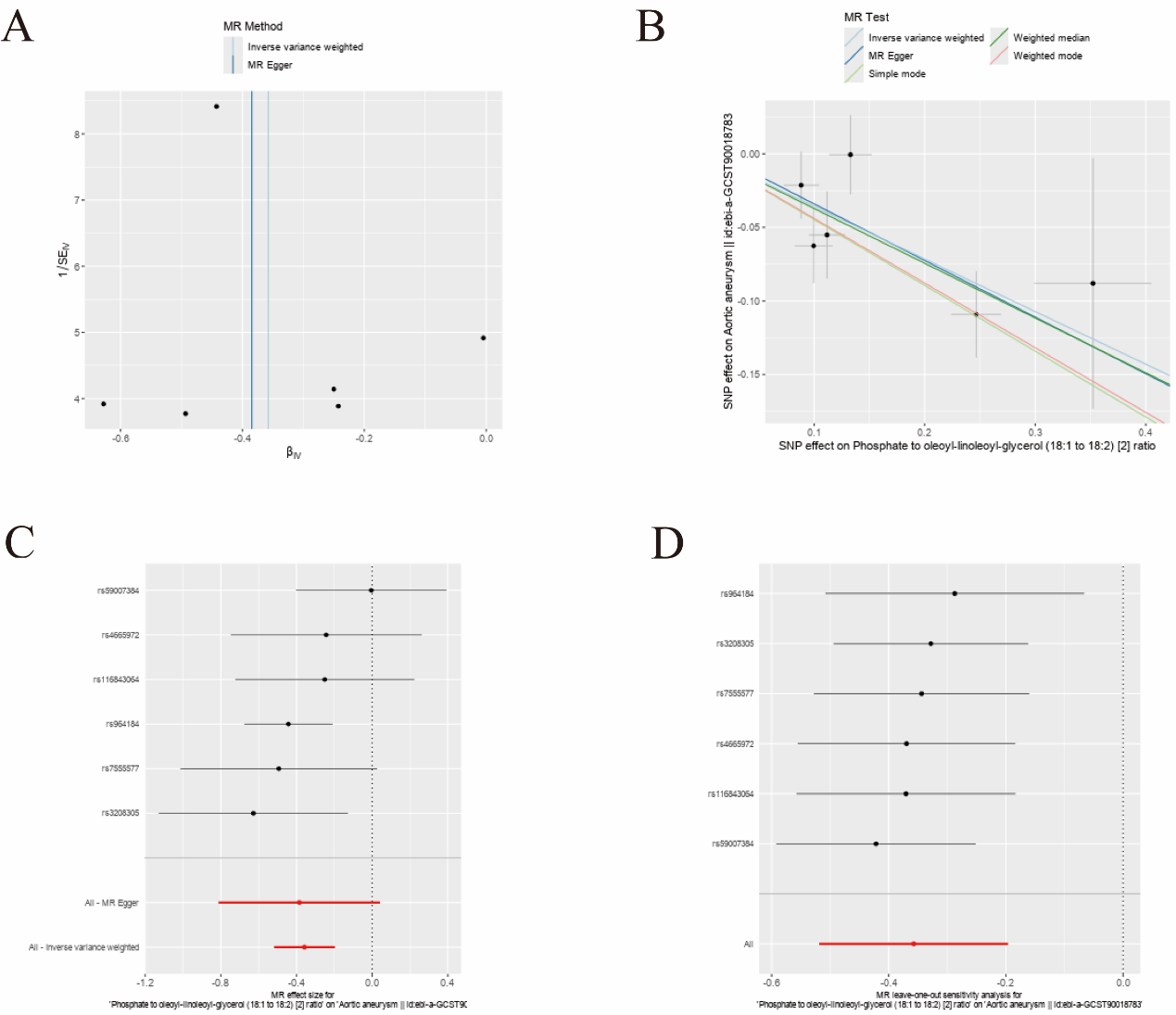


Supplementary Figure 26. The distribution of SNPs and sensitivity analyses for the causal effect of Phosphate to oleoyl-linoleoyl-glycerol (18:1 to 18:2) [2] ratio on AAA. A, The funnel plot for the distribution of SNPs. B, Scatter plot for the causal effect. C, Forest plot of single SNP MR. D, Forest plot of leave-one-out sensitivity analysis.


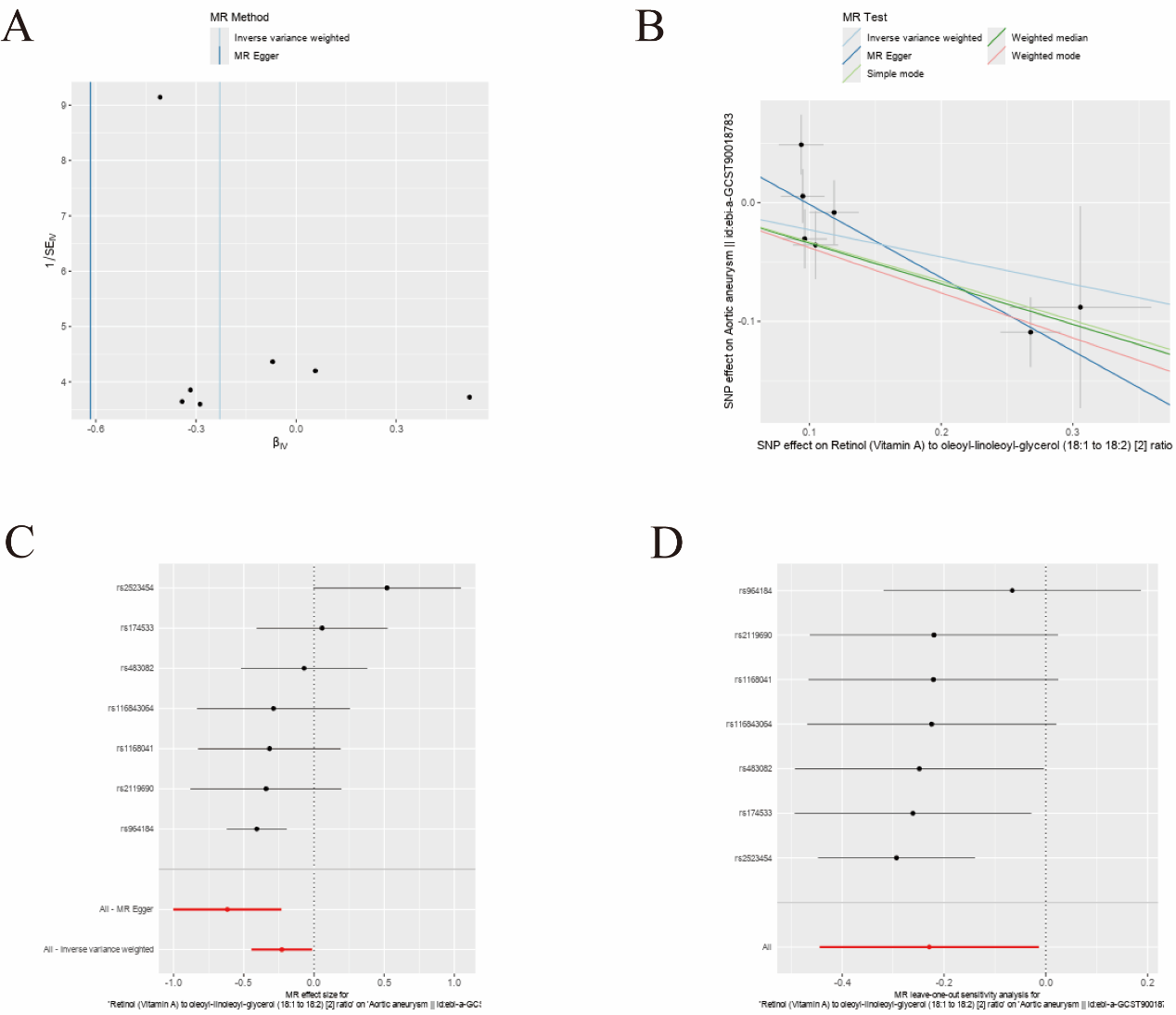


Supplementary Figure 27. The distribution of SNPs and sensitivity analyses for the causal effect of Retinol (Vitamin A) to oleoyl-linoleoyl-glycerol (18:1 to 18:2) [2] ratio on AAA. A, The funnel plot for the distribution of SNPs. B, Scatter plot for the causal effect. C, Forest plot of single SNP MR. D, Forest plot of leave-one-out sensitivity analysis.


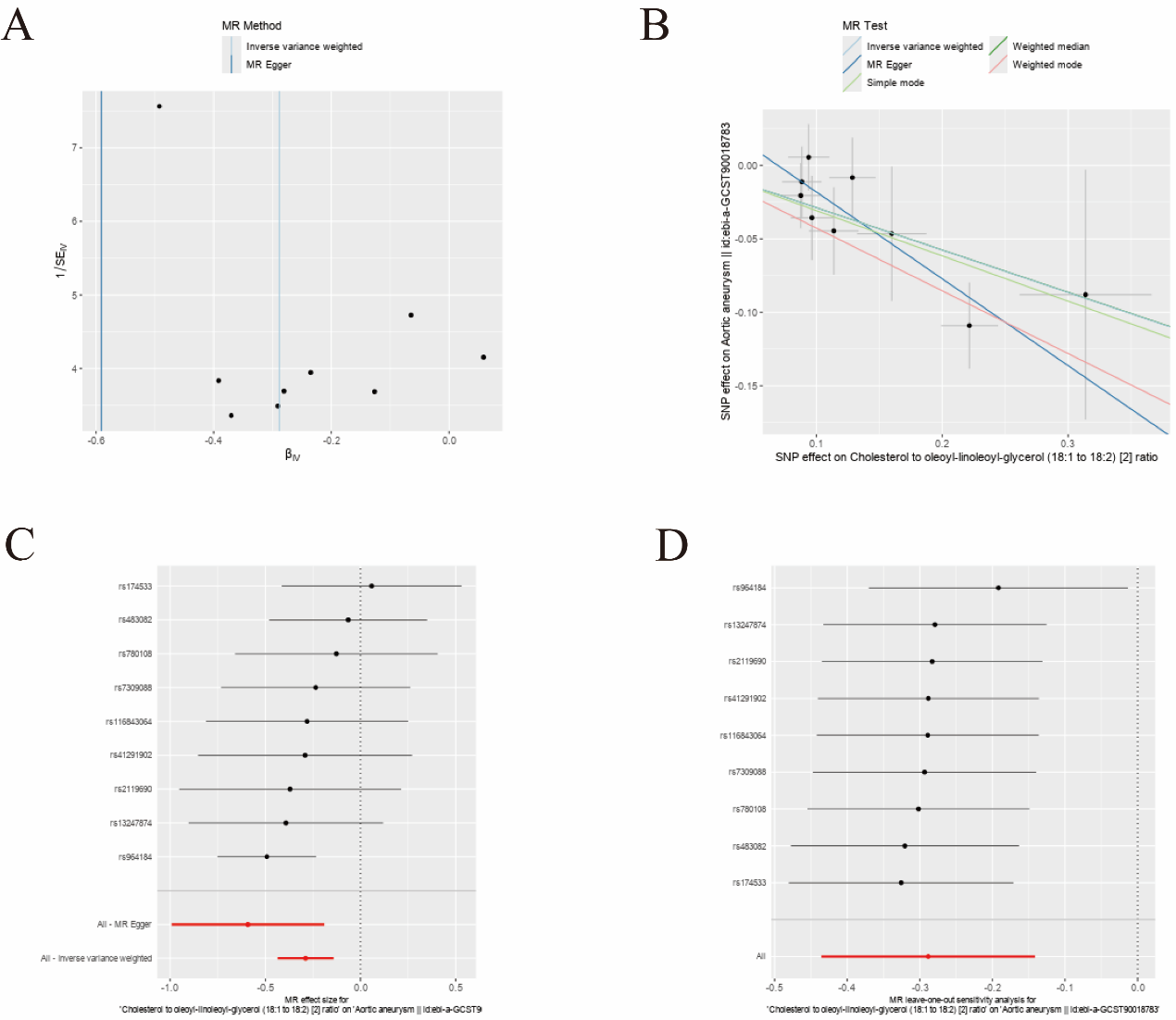


Supplementary Figure 28. The distribution of SNPs and sensitivity analyses for the causal effect of Cholesterol to oleoyl-linoleoyl-glycerol (18:1 to 18:2) [2] ratio on AAA. A, The funnel plot for the distribution of SNPs. B, Scatter plot for the causal effect. C, Forest plot of single SNP MR. D, Forest plot of leave-one-out sensitivity analysis.


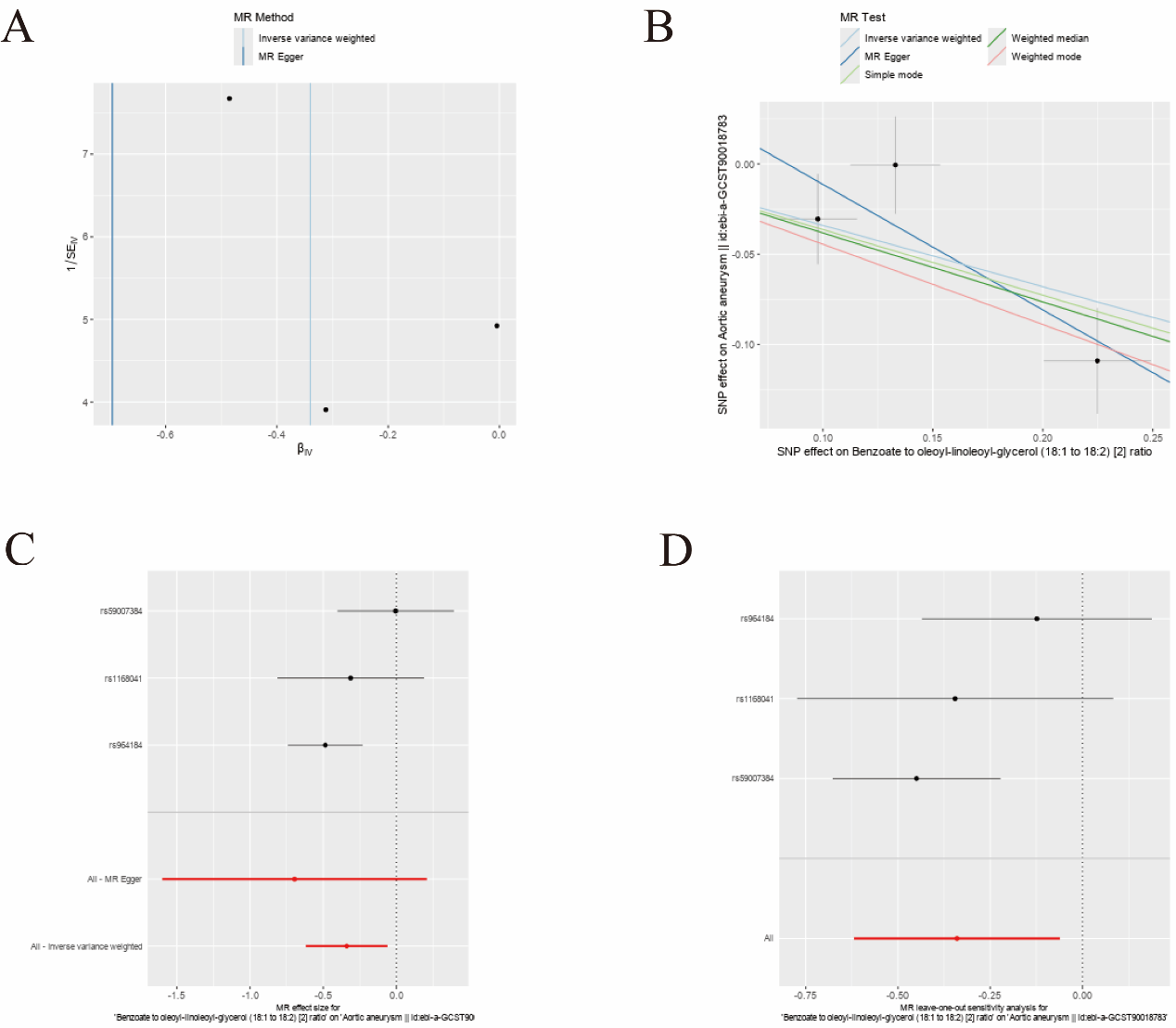


Supplementary Figure 29. The distribution of SNPs and sensitivity analyses for the causal effect of Benzoate to oleoyl-linoleoyl-glycerol (18:1 to 18:2) [2] ratio on AAA. A, The funnel plot for the distribution of SNPs. B, Scatter plot for the causal effect. C, Forest plot of single SNP MR. D, Forest plot of leave-one-out sensitivity analysis.
